# Computerized clinical decision support systems for acute care management: A decision-maker-researcher partnership systematic review of effects on process of care and patient outcomes

**DOI:** 10.1186/1748-5908-6-91

**Published:** 2011-08-03

**Authors:** Navdeep Sahota, Rob Lloyd, Anita Ramakrishna, Jean A Mackay, Jeanette C Prorok, Lorraine Weise-Kelly, Tamara Navarro, Nancy L Wilczynski, R Brian Haynes

**Affiliations:** 1 College of Medicine, University of Saskatchewan, 107 Wiggins Road, Saskatoon, SK, Canada; 2 Department of Pediatrics, McMaster University, 1280 Main Street West, Hamilton, ON, Canada; 3 Hamilton Health Sciences, 1200 Main Street West, Hamilton, ON, Canada; 4 McMaster University, 1280 Main Street West, Hamilton, ON, Canada; 5 Health Information Research Unit, Department of Clinical Epidemiology and Biostatistics, McMaster University, 1280 Main Street West, Hamilton, ON, Canada; 6 Department of Medicine, McMaster University, 1280 Main Street West, Hamilton, ON, Canada

## Abstract

**Background:**

Acute medical care often demands timely, accurate decisions in complex situations. Computerized clinical decision support systems (CCDSSs) have many features that could help. However, as for any medical intervention, claims that CCDSSs improve care processes and patient outcomes need to be rigorously assessed. The objective of this review was to systematically review the effects of CCDSSs on process of care and patient outcomes for acute medical care.

**Methods:**

We conducted a decision-maker-researcher partnership systematic review. MEDLINE, EMBASE, Evidence-Based Medicine Reviews databases (Cochrane Database of Systematic Reviews, DARE, ACP Journal Club, and others), and the Inspec bibliographic database were searched to January 2010, in all languages, for randomized controlled trials (RCTs) of CCDSSs in all clinical areas. We included RCTs that evaluated the effect on process of care or patient outcomes of a CCDSS used for acute medical care compared with care provided without a CCDSS. A study was considered to have a positive effect (*i.e.*, CCDSS showed improvement) if at least 50% of the relevant study outcomes were statistically significantly positive.

**Results:**

Thirty-six studies met our inclusion criteria for acute medical care. The CCDSS improved process of care in 63% (22/35) of studies, including 64% (9/14) of medication dosing assistants, 82% (9/11) of management assistants using alerts/reminders, 38% (3/8) of management assistants using guidelines/algorithms, and 67% (2/3) of diagnostic assistants. Twenty studies evaluated patient outcomes, of which three (15%) reported improvements, all of which were medication dosing assistants.

**Conclusion:**

The majority of CCDSSs demonstrated improvements in process of care, but patient outcomes were less likely to be evaluated and far less likely to show positive results.

## Background

Computerized clinical decision support systems (CCDSSs) are information systems intended to improve clinical decision-making. CCDSSs match individual patient data to a computerized knowledge base that uses software algorithms to generate patient-specific recommendations that are delivered to healthcare practitioners [1-3].

This review, acute medical care, is one of a series of six on specific interventions of CCDSSs, including primary preventive care, chronic disease management, diagnostic test ordering, drug prescribing and management, and therapeutic drug monitoring and dosing. The review process involved senior healthcare managers in setting priorities and co-sponsoring the review process with an academic review team, and engagement of key clinical leaders in each review to establish review questions, guide data extraction needed for clinical application, and draw conclusions from a practical clinical perspective [4].

Expectations are high for the utility of CCDSSs in acute care because acute care in hospitals and emergency rooms is the most intensive and expensive part of the healthcare system on a per patient basis, but many concerns and problems have been identified [5]. As with any healthcare intervention, CCDSSs purporting to improve patient care or outcomes should be rigorously evaluated before being routinely implemented in clinical practice [6]. This systematic review focuses on the use of CCDSSs for management of medical problems in acute care settings and summarizes the most rigorous evidence to date concerning the effects of CCDSSs in acute medical care. An example of such a CCDSS includes advice for paramedics responding to emergency calls.

## Methods

Methods for this review are described in detail elsewhere [4]http://www.implementationscience.com/content/5/1/12 with pertinent details provided here.

### Research question

Do CCDSSs improve process of care or patient outcomes for acute medical care?

### Partnering with decision makers

The review was conducted using a partnership model [4] with 2 main groups: decision makers from local health institutions and research staff of the Health Information Research Unit (HIRU) at McMaster University. There were two types of decision makers--senior health managers of Hamilton Health Sciences (a large academic health sciences centre) provided overall guidance and endorsement, and a clinical service leader (RL, a pediatrician) provided specific guidance for acute care management. HIRU research staff and students were responsible for completing the literature search, and appraising, extracting, and synthesizing the data. The goal of the partnership model was to maximize knowledge translation with respect to potential local CCDSS implementation.

### Search strategy

**Figure 1:**
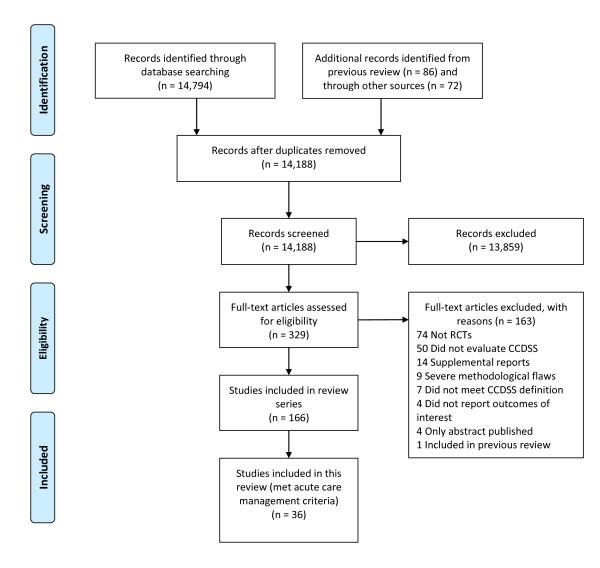
Flow diagram of included and excluded studies for the update 1 January 2004 to 6 January 2010 with specifics for acute care management*. *Details provided in: Haynes RB *et al. *[4]. Two updating searches were performed, for 2004 to 2009 and to 6 January 2010 and the results of the search process are consolidated here.

We reassessed all citations in our most recent review [3] and retrieved new citations from that review's September 2004 closing date to 6 January 2010, in all languages, by employing a comprehensive search strategy of MEDLINE, EMBASE, Evidence-Based Medicine Reviews databases (Cochrane Database of Systematic Reviews, DARE, ACP Journal Club, and others), and the Inspec bibliographic database. Pairs of reviewers independently evaluated each citation and abstract to determine the eligibility of all studies identified in our search. Disagreements were resolved by a third reviewer or by consensus. Inter-reviewer agreement on study eligibility was measured using the unweighted Cohen's kappa (κ), and was excellent (κ = 0.93; 95% confidence interval [CI], 0.91 to 0.94) overall. A panel of reviewers--including a physician, a pharmacist, and two individuals trained in health research methods--reviewed eligible studies and assigned them to appropriate care area(s). Acute care referred to episodic health conditions that could be possibly cured or stabilised in less than six months. Figure 1 summarizes the study selection process, including specifics for acute care management.

Several studies addressed two or more clinical care areas; the review for each care area focused only on the study outcomes that were most relevant for that area. Most study overlaps for acute care were with therapeutic drug monitoring and drug prescribing.

Six studies were excluded from acute care after the initial selection. Three studies met initial criteria but were later excluded for confounding of healthcare provider across treatment groups (*e.g.*, pharmacist using CCDSS versus physician giving usual care) [7-9]. Three studies that met review criteria did not report relevant data for acute care conditions [10-12].

### Study selection

Studies were included if they met all of the following five criteria: evaluated a CCDSS used for acute care; used an randomized controlled trial (RCT) design where patient care with a CCDSS was compared to patient care without a CCDSS; assessed effects among healthcare professionals in clinical practice or post-graduate training; provided patient-specific information in the form of assessments (management options or probabilities) or recommendations to the clinicians, who remained responsible for actual decisions; and measured clinical performance (a measure of process of care) or patient outcomes (including any aspect of patient well-being). Studies were excluded if they provided only summaries of patient information, feedback on groups of patients without individual assessment, or only computer-aided instruction; used simulated patients; or used CCDSSs for image analysis.

### Data extraction

Pairs of reviewers independently extracted the following data from all eligible studies: study setting, study methods, CCDSS characteristics, patient characteristics, and outcomes. Disagreements were resolved by a third reviewer or by consensus. We attempted to contact primary authors of all 36 included studies and 28 authors (78%) replied and confirmed data, including six who had previously replied and confirmed data in our most recent review [3].

### Assessment of study quality

Methodological quality was evaluated using a 10-point scale consisting of five potential sources of bias, and based on an extension of the Jadad scale [13]. A score of 10 on the scale indicated the highest study quality [4].

### Assessment of CCDSS intervention effects

Studies with multiple treatment arms were counted as a positive study if any of the treatment arms showed a benefit over the control arm. Outcomes were considered primary if reported by the author as 'primary' or 'main' outcomes. If no primary outcomes were reported and a power statement was provided, then the outcome on which the power statement was based was considered primary. The use of effect sizes was judged to be inappropriate because of the high degree of heterogeneity in almost every aspect of the individual studies. Effects for each CCDSS were evaluated based on relevant outcomes showing a statistically significant difference (2*p* <0.05). Effects were identified as statistically significantly positive (+) or negative (-), or no effect (0), based on the following predefined hierarchy of outcomes:

1. If a single primary outcome was reported, in which all components were applicable to acute medical care, this was the only outcome evaluated.

2. If >1 primary outcome was reported, only the applicable primary outcomes were evaluated, and judged positive if ≥50% were statistically positive.

3. If no primary outcomes were reported (or only some of the primary outcome components were relevant) but overall analyses were provided, the overall analyses were evaluated as primary outcomes. Subgroup analyses were not considered.

4. If no primary outcomes or overall analyses were reported, or only some components of the primary outcome were relevant for the care area, any reported applicable pre-specified outcomes were evaluated.

5. If no clearly pre-specified outcomes were reported, any available relevant outcomes were considered.

6. If statistical comparisons were not reported, 'effect' was designated as not evaluated (denoted as ...).

These criteria are more specific than those used in our previous review; therefore, the assignment of effect was adjusted for some studies included in the earlier review.

### Data synthesis and analysis

We summarized data and p-values reported in individual studies. CCDSS characteristics were analyzed and interpreted with the study as the unit of analysis. Data were summarised using descriptive summary measures, including proportions for categorical variables and means (± standard deviation [SD]) for continuous variables. All analyses were carried out using SPSS v.15. A 2-sided *p *< 0.05 indicated statistical significance.

A sensitivity analysis was conducted to assess the possibility of biased results in studies with a mismatch between the unit of allocation (*e.g.*, clinicians) and the unit of analysis (*e.g.*, individual patients without adjustment for clustering). Success rates comparing studies with matched and mismatched analyses were compared using chi-square for comparisons. No differences in reported success were found for either process of care outcomes (Pearson X^2 ^= 2.70, 2p = 0.10) or patient outcomes (Pearson X^2 ^= 0.39, 2p = 0.53). Accordingly, results have been reported without distinction for mismatch.

## Results

Re-examination of the articles included in the prior review [3] yielded 20 articles that met our criteria for acute care [14-33]. From the current update (1 January 2004 to 6 January 2010), we screened 11,790 citations for all CCDSS interventions, retrieved 243 full-text articles, and determined that 16 new studies [34-50] met our criteria for acute care (Figure 1), for a total of 36 studies described in 37 articles, published from 1984 to 2009 [14-50]. Twenty-six included studies contribute outcomes to this review as well as other CCDSS interventions in the series; one study [26] to four reviews, four studies [19,25,41,47] to three reviews, and 21 studies [14,16-18,21,22,24,27-32,34-36,40,42,48-50] to two reviews; but we focused here on acute care-relevant outcomes.

Summary of trial quality is reported in Additional file 1, Table S1; system characteristics in Additional file 2, Table S2; study characteristics in Additional file 3, Table S3; outcome data in Additional file 4, Table S4 and Table 1; and other CCDSS-related outcomes in Additional file 5, Table S5.

**Table 1:** Results for CCDSS trials of acute care

Study	Methods Score	Indication	No. of centres/providers/patients	Process of care outcomes	CCDSS Effect^a^	Patient outcomes	CCDSS Effect ^a^
**Management Assistants - Alerts and Reminders**							

Terrell, 2009 [48]	9	CCDSS provided alerts to avoid inappropriate prescriptions in geriatric outpatients during discharge from emergency care.	1/63*/5,162'	ED visits by older adults that resulted in prescriptions for ≥1 of nine targeted inappropriate medications.	**+**	...	**...**
Peterson, 2007 [36]^b^	4	CCDSS provided dosing advice for high-risk drugs in geriatric patients in a tertiary care academic health centre.	1/778/2,981*	Ratio of prescribed to recommended doses.	**+**	...	**...**
Kroth, 2006 [39]	7	CCDSS identified low temperature values and generated prompts to repeat measurement in order to improve accuracy of temperature capture by nurses at the bedside of non-critical care hospital patients.	.../337*/90,162	Low temperatures recorded by nursing personnel type.	**+**	...	**...**
Rood, 2005 [34]	8	CCDSS recommended timing for glucose measurements and administration of insulin in critically ill patients.	1/104/484*	Deviation between advised and actual glucose measurement times; Time that patients' glucose levels were within specified range over 10 weeks; Adherence to guideline for timing of glucose measurement.	**+**	...	**...**
Zanetti, 2003 [47]	8	CCDSS provided alarm and alert for redosing of prophylactic antibiotics during prolonged cardiac surgery.	1/.../447*	Intraoperative redose of antibiotics.	**+**	Surgical-site infection.	**0**
Selker, 2002 [29]	8	CCDSS generated recommendations for management of thrombolytic and other reperfusion therapy in acute myocardial infarction.	28/.../1,596*	Detection of ST-segment elevation without AMI; Receipt of thrombolytic therapy; Receipt of thrombolytic therapy and contraindications; Treatment of patients with AMI.	**0**	Mortality; Stroke; Thrombolysis-related bleeding events requiring transfusion.	**0**
Dexter, 2001 [19]	10	CCDSS provided guideline-based reminders for preventive therapies in hospital inpatients.	...*/202/3,416	Hospitalizations with an order for therapy; Hospitalizations during which therapy was ordered for an eligible patient.	**+**	...	**...**
Kuperman, 1999 [23]	4	CCDSS detected critical laboratory results for all medical and surgical inpatients and alerted health provider that the results were ready.	1/.../...*	Length of time interval from filing alerting result to ordering of appropriate treatment; Filing time and resolution of critical condition.	**+**	Adverse events within 48 hours of alert.	**0**
Overhage, 1997 [26]	8	CCDSS identified corollary orders to prevent errors of omission for any of 87 target tests and treatments in hospital inpatients on a general medicine ward.	1*/92/2,181	Compliance with corollary orders; Pharmacist intervention with physicians for significant errors.	**+**	LOHS; Serum creatinine level.	**0**
Overhage, 1996 [25]	10	CCDSS provided reminders of 22 US Preventive Services Task Force preventive care measures for hospital inpatients, including cancer screening, preventive screening and medications, diabetes care reminders, and vaccinations.	1*/78/1,622	Compliance with preventive care guidelines.	**0**	...	**...**
White, 1984 [31]	4	CCDSS identified concerns (drug interactions or signs of potential digoxin intoxication) in inpatients taking digoxin.	1/.../396*	Physician actions related to alerts.	**+**	...	**...**

**Management Assistants - Guidelines and algorithms**							

Helder, 2008 [43]	6	CCDSS generated recommendations for management of incubator settings in neonatal ICU.	1/117/136*	Days to regain birthweight.	**0**	Intraventricular haemorrhage; Sepsis; Mortality.	**0**
Davis, 2007 [42]	9	CCDSS provided evidence-based data relating to appropriate prescribing for upper respiratory tract infections in paediatric outpatients.	2/44*/12,195	Prescriptions consistent with evidence-based recommendations.	**+**	...	**...**
Rothschild, 2007 [37,38]	7	CCDSS generated recommendations for non-emergent inpatient transfusion orders.	1/1,414*/3,903	Appropriateness ratings of decision support interventions.	**+**	Severely undertransfused patients.	**0**
Kuilboer, 2006 [41]	10	CCDSS assisted monitoring and treatment of asthma and COPD in daily practice in primary care.	32*/40/156,772	Contacts; Peak total flow; Peak flow ratio; FEV1; FEV1 ratio measurements; Antihistamines prescriptions; Cromoglycate prescriptions; Deptropine prescriptions; Oral bronchodilators prescriptions; Oral corticosteroids prescriptions.	**0**	...	**...**
Paul, 2006 [40]	10	CCDSS assisted management of antibiotic treatment in hospital inpatients.	15*/.../2,326	Appropriate antibiotic treatment.	**+**	Duration of hospital; Duration of fever; Mortality.	**0**
Brothers, 2004 [46]	6	CCDSS provided recommendations for surgical management of patients with peripheral arterial disease.	2/3/206*	Agreement between surgeon's initial and final treatment plan.	**0**	...	**...**
Hamilton, 2004 [44]	8	CCDSS provided evaluation and recommendations of labour progress and need for caesarean sections.	7/.../4,993*	Caesarean sections.	**0**	Recorded indication of dystocia; Apgar score.	**0**
Hales, 1995 [20]	4	CCDSS evaluated appropriateness of inpatient admissions.	1/.../1,971*	Unnecessary hospital admissions.	**0**	...	**...**
Wyatt, 1989 [33]	5	CCDSS generated recommendations resulting in identification of high-cardiac risk patients among patients with chest pain attending the ED.	1/15/153*	Overall management accuracy; Time until cardiac care unit admission.	**...**	...	**...**

**Diagnostic Assistants**							

Roukema, 2008 [35]	6	CCDSS provided advice for the diagnostic management for children with fever without apparent source in the ED.	1/15/164*	Test ordering.	**+**	Time spent at ED.	**0**
Stengel, 2004 [45]	8	CCDSS assisted electronic documentation of diagnosis and findings in patients admitted to orthopaedic ward.	1/6/78*	Diagnoses per patient.	**+**	...	**...**
Bogusevicius2002 [15]	7	CCDSS generated diagnosis of acute SBO in surgical inpatients.	1/.../80	Diagnosis of acute SBO; Diagnosis of partial SBO; Time to diagnosis.	**0**	Bowel necrosis; Morbidity; Mortality; LOHS; Proportion of patients receiving each type of surgical procedure: open lysis of adhesion; laparoscopic lysis of adhesion; bowel resection.	**0**
**Medication Dosing Assistants**							

Cavalcanti, 2009 [49]	8	CCDSS recommended insulin dosing and glucose monitoring to achieve glucose control in patients in ICU.	5/60/168*	BG measurements obtained per patient; Time with BG controlled.	**+**	BG during ICU stay; Hypoglycaemia.	**+/0**
Saager, 2008 [50]	6	CCDSS recommended insulin dosing and glucose assessment frequency for diabetic patients in cardiothoracic ICU.	1/.../40*	BG in range (90 to 150 mg/dL); Time in range.	**+**	Mean BG; Mean time to BG<150 mg/dL.	**+**
Peterson, 2007 [36]^b^	4	CCDSS provided dosing advice for high-risk drugs in geriatric patients in a tertiary care academic health centre.	1/778/2,981*	Ratio of prescribed to recommended doses.	**+**	...	**...**
Poller, 1998 [28]	3	CCDSS provided dosing for oral anticoagulants in outpatients with AF, DVT or PE, mechanical heart valves, or other indications.	5/.../285*	Time within target INR range for all patients and all ranges; Proportion of time in target range.	**+**	...	**...**
Vadher, 1997 [30]	6	CCDSS provided dosing recommendations for warfarin initiation and maintenance for inpatients and outpatients with DVT, PE or systemic embolus, AF, valve disease, or mural thrombus, or who needed prophylaxis.	1/49/148*	Time to reach therapeutic range; Time to reach stable dose; Time to first pseudoevent; Days at INR 2 to 3.	**0**	Mortality; Haemorrhage events; Thromboembolism events.	**...**
Casner, 1993 [18]	3	CCDSS predicted theophylline infusion rates for inpatients with asthma or COPD.	1/.../47*	Serum theophylline levels; Absolute difference between final and target theophylline levels; Mean difference between target and mean final theophylline level; Subtherapeutic final theophylline levels; Toxic final theophylline levels.	**0**	Theophylline-associated toxicity; LOHS; Duration of treatment.	**0**
Burton, 1991 [16]	6	CCDSS provided aminoglycoside dosing for inpatients with clinical infections.	1*/.../147	Beginning aminoglycoside dose; Ending aminoglycoside dose; Ending aminoglycoside dose interval; Peak aminoglycoside level; Peak aminoglycoside level >4 mg/L; Trough aminoglycoside levels; Proportion of patients with trough aminoglycoside levels ≥2 mg/L; Length of aminoglycoside therapy.	**0**	Proportion of patients cured; Response to therapy; Treatment failure; Mortality; Indeterminate response; Nephrotoxicity; LOHS; LOHS after start of antibiotics.	**0**
Begg, 1989 [14]	4	CCDSS provided individualised aminoglycoside dosing for inpatients receiving gentamicin or tobramycin.	.../.../50*	Achievement of peak and trough aminoglycoside levels.	**+**	Mortality; Creatinine clearance during therapy.	**0**
Gonzalez, 1989 [22]	5	CCDSS estimated aminophylline loading and maintenance dosing for ED patients.	.../.../67*	Aminophylline loading dose to achieve target serum theophylline level; Aminophylline maintenance dose to achieve target serum theophylline level; Theophylline level.	**+**	Discharged from ED within 8 hours; Adverse effects; Peak flow rate throughout the study.	**0**
Hickling, 1989 [21]	3	CCDSS provided dosing and dose intervals of aminoglycoside in critically ill patients.	1/.../32*	Proportion of patients outside of therapeutic range; Peak plasma aminoglycoside levels; Trough levels; Proportion of patients with 48-72 h peak plasma levels.	**+**	Estimated creatinine clearance during recovery.	**0**
Carter, 1987 [17]	2	CCDSS provided dosing recommendations for warfarin initiation and adjustments in hospital inpatients.	1/.../54*	Days from administration of first warfarin dose to achievement of stabilization dosage.	**0**	Time to discharge.	**..**.
White, 1987 [32]	6	CCDSS provided dosing recommendations for warfarin therapy in patients hospitalized with DVT, cerebrovascular accident, transient ischemic attack, PE or AF.	2/.../75*	Time to reach a stable therapeutic dose; Time to reach a therapeutic PR ratio; Patients with PR above therapeutic range during hospital stay; Predicted/observed PR; Absolute error.	**+**	LOHS; In-hospital bleeding complications.	**+**
Hurley, 1986 [24]	8	CCDSS provided dosing for theophylline in inpatients with acute air-flow obstruction.	1/.../96*	Theophylline levels above therapeutic range; Theophylline levels below therapeutic range; Trough theophylline levels in therapeutic range during oral therapy; Serum theophylline levels; 1st serum level during oral therapy; Trough levels during oral therapy.	**0**	Peak expiratory flow rate; Air flow obstruction symptoms; Side effects; Mortality.	**0**
Rodman, 1984 [27]	6	CCDSS recommended lidocaine dosing for patients in intensive or coronary care units.	1/.../20*	Plasma lidocaine levels in middle of therapeutic range.	**+**	Toxic response requiring lidocaine discontinuation or dosage reduction.	**0**

Abbreviations: AF, atrial fibrillation; AMI, acute myocardial infarction; BG, blood glucose; CCDSS, computerized clinical decision support system; COPD, chronic obstructive pulmonary disease; DVT, deep vein thrombosis; ED, emergency department; FEV1, forced expiratory volume in 1 second; ICD, International Classification of Diseases; ICU, intensive care unit; INR, international normalised ratio; LOHS, length of hospital stay; PE, pulmonary embolus; PR, prothrombin ratio; SBO, small bowel obstruction.

*Unit of allocation.

^a^Outcomes are evaluated for effect as positive (+) or negative (-) for CCDSS, or no effect (0), based on the following hierarchy. An effect is defined as ≥50% of relevant outcomes showing a statistically significant difference (2*p *< 0.05):

1. If a single primary outcome is reported, *in which all components are applicable*, this is the only outcome evaluated.

2. If >1 primary outcome is reported, the ≥50% rule applies and only the primary outcomes are evaluated.

3. If no primary outcomes are reported (or only some of the primary outcome components are relevant) but overall analyses are provided, the overall analyses are evaluated as primary outcomes. Subgroup analyses are not considered.

4. If no primary outcomes or overall analyses are reported, or only some components of the primary outcome are relevant for the application, any reported prespecified outcomes are evaluated.

5. If no clearly prespecified outcomes are reported, any available outcomes are considered.

6. If statistical comparisons are not reported, 'effect' is designated as not evaluated (...).

^b^Study included in two categories.

### Study quality

Based on the 10-point scale for methodological quality, the mean score was 6.4 (95% CI 5.7 to 7.2), with a range from 2 to 10 (see Additional file 1, Table S1). However, the quality of studies increased over time: the mean score was 5.6 (4.6 to 6.6) for the 20 studies from the 2005 review compared with 7.5 (6.7 to 8.3) for the 16 studies retrieved after 2005 (*p *= 0.01). Fifty-eight percent (21/36) of studies concealed study group allocation before randomization [15,19,23-27,29,33,34,37-42,44-49], and 28% (10/36) of studies employed cluster randomization by practice or physician [16,19,25,26,37-42,48].

### CCDSS and study characteristics

Additional file 2, Table S2 describes key characteristics of the included CCDSSs. Denominators vary because not all trials reported on all features considered. The CCDSSs were pilot tested in 63% (19/30) of studies [15,17,19,24,27-30,33,34,36-42,45,46,49], users were trained in the CCDSSs at the time of implementation in 56% (18/32) of studies [17,18,22,23,27,28,31-36,39,41,42,45,46,49], 97% (33/34) of CCDSSs provided feedback at the time of patient care [14-16,18,19,21-23,25-50], 97% (34/35) of CCDSSs suggested diagnoses/treatment/procedures [14-19,21-43,45-50], and 76% (25/33) of the study authors were also the developers of the CCDSSs [14,15,19,21,23,25-27,30-42,45-49]. Most studies did not report the interface details for the CCDSSs.

In 59% (20/34) of the studies, the CCDSSs were stand alone systems [14-18,21,22,24,27-30,32,33,35,40,45,46,49,50], and in 38% (13/34) of the studies, the CCDSS was integrated with a computerized order entry and/or an electronic medical record system [19,20,23,25,26,31,34,36-39,41,42,47,48]. The source of data entry was apparent in 86% (31/36) of the studies [14,17-20,23-49]. Data entry was automated via the electronic medical record system in only 29% (9/31) of cases [19,23,25,26,31,34,41,42,47]. The majority (74%, 23/31) used manual data entry (decision-maker [14,18,26-28,32,36-39,45,46,48], 39% (12/31); existing staff [17,19,20,24,29,32,35,47], 26% (8/31); project staff [19,30,40,43-45], 19% (6/31); patient [46], 3% (1/31)). The methods for delivery of the recommendation were clear in 81% (29/36) of the studies, with the most common method of delivery being through a desktop/laptop computer [19,23,25,26,28,32,34-43,47-49] (62%, 18/29). Most CCDSSs had multiple user groups: 78% (28/36) were physicians [14-22,24-29,31,32,34-38,40-42,45-48], 47% (17/36) were trainees [16,17,19,22,23,25,26,31-33,36-39,42,43,45,48], 19% (7/36) were advanced practice nurses [30,35,36,39,43,46,47], 8% (3/36) were pharmacists [27,32,36], and 22% (8/36) were other health professionals [20,33,34,39,42,44,49,50].

Eligible studies were conducted in 121 different clinics at 106 sites, involving over 3,417 healthcare practitioners and 202,491 patients (see Additional file 3, Table S3). Fifty-three percent (19/36) of studies were missing data on the number of practitioners [14-18,20-24,27-29,31,32,40,44,47,50], 6% (2/36) were missing data on the number of patients [23,39], and 11% (4/36) were missing data on both the number of clinics and sites [14,19,22,39]. Some of the 36 studies were conducted in more than one country, but most studies were conducted in the United States [16-20,22,23,25-27,29,31,32,36-39,42,44,46-48,50] (61%, 22/36), followed by the Netherlands [34,35,41,43] (11%, 4/36), the United Kingdom [28,30,33] (8%, 3/36), Germany [40,45] and New Zealand [14,21] (6% each, 2/36), and Australia [24], Brazil [49], Canada [44], Denmark [28], Israel [40], Italy [40], Lithuania [15], Norway [28], and Portugal [28] (3% each, 1/36). Fifty-eight percent of the studies reported solely public funding [16,17,19,23,25-30,33,35,37-42,46-48,50], 8% (3/36) reported solely private funding [21,22,36], 6% (2/36) reported both private and public funding [24,49], and 28% (10/36) did not report their funding source [14,15,18,20,31,32,34,43-45].

### CCDSS effectiveness

Table 1 provides a summary of the effect of CCDSSs on process of care and patient outcomes (detailed outcome information is provided in Additional file 4, Table S4). Among studies that reported sufficient data for analysis, 63% (22/35) reported an improvement in process of care outcomes [14,19,21-23,26-28,31,32,34-40,42,45,47-50] and 15% (3/20) reported an improvement in patient outcomes [32,49,50]. One of the studies that reported an improvement in process of care outcomes, Cavalcanti 2009, had two patient outcomes: one showed a benefit with CCDSS for blood sugar control compared with conventional care, but at the expense of increased hypoglycemic episodes.

Studies could be organised into four separate categories, management assistants--alerts/reminders, management assistants--guidelines/algorithms, diagnostic assistants, and medication dosing assistants, with only one study [36] falling into two categories.

### Management assistants - alerts and reminders

Eleven trials tested a management assistant using alerts and reminders, such as alerting pharmacists to possible drug interactions [29,31,36,47,48] or giving reminders to physicians for preventive therapies like vaccines [19,23,25,26,34,39]. Nine of the 11 trials (82%) that evaluated process of care outcomes demonstrated an improvement [19,23,26,31,34,36,39,47,48], and none of four studies assessing patient outcomes showed improvement.

The studies of highest quality in this group all produced improvements in process of care outcomes. Overhage *et al. *tested a CCDSS that generated corollary orders to prevent errors of omission for any of 87 target tests and treatments in hospital inpatients [26]. In comparison to a computerized order entry system alone, compliance with corollary orders was increased in the CCDSS group and the number of pharmacist interventions with physicians for significant errors was decreased. Another high-quality study, Dexter *et al*., gave reminders for preventive therapies in hospital inpatients and showed an increase in the proportion of eligible hospitalized patients who received the targeted preventive therapy [19]. Terrell *et al. *assessed a CCDSS that provided alerts to avoid inappropriate prescriptions in geriatric outpatients during discharge from emergency care [48]. Inappropriate medication prescriptions decreased in the CCDSS group when compared to usual care. Kroth *et al. *had the largest patient population in this group (N = 90,162) and tested a CCDSS which helped improve the accuracy of temperature capture by nurses for non-critical care hospital patients [39]. The study reported a decrease in the number of (presumed erroneous) low temperatures recorded by nurses in the CCDSS group compared to usual care. Kuperman *et al. *tested a CCDSS that notified health providers when critical laboratory results for all medical and surgical inpatients were ready [23]. In comparison to usual care, the CCDSS group reduced the time from recording the alert to ordering the appropriate treatment. Zanetti *et al. *provided alerts for redosing of prophylactic antibiotics during prolonged cardiac surgery and showed an increase in the number of intraoperative redoses compared to usual care [47]. The CCDSS in White *et al. *identified signs and risk factors for digoxin intoxication for inpatients [31]. The trial reported an increase in the number of physician actions related to the alerts in the CCDSS group compared to usual care. Rood *et al. *developed a guideline for tight glycaemic control in intensive care unit (ICU) patients and compared a CCDSS version to the paper-based system [34]. Use of the CCDSS resulted in stricter adherence to the guideline, both in terms of timing of glucose measurements and use of advised insulin doses. This resulted in a small improvement in patient glycaemic control; however, the improvement was judged to be not clinically important.

### Management assistants - guidelines and algorithms

Nine studies [20,33,37,38,40-44,46] examined a management assistant employing guidelines and algorithms--these CCDSSs generated recommendations for the management of acute health issues using guidelines or algorithms, such as evidence-based electronic prescribing in paediatric care [42]. Of the eight studies that assessed process of care outcomes, three (38%) demonstrated improvements [37,38,40,42], and none of the four studies that assessed patient outcomes showed an improvement.

Process improvements occurred in a multicentre study of high methodological quality by Paul *et al. *[40]. The CCDSS assisted with choice of empiric antibiotic treatment in hospital inpatients and improved appropriate antibiotic therapy in comparison to usual care. Davis *et al. *assessed appropriate prescribing for upper respiratory tract infections in paediatric outpatients [42]. Compared to usual care, the CCDSS increased prescriptions that were consistent with evidence-based recommendations. Rothschild *et al. *tested a CCDSS that produced recommendations for non-emergent inpatient transfusion orders, and showed improvement in guideline adherence as measured by the percentage of appropriate and inappropriate transfusion orders [37,38]. The methodologically sound study by Kuilboer *et al. *had the largest patient population of all the acute care trials (N = 156,772), but did not report any improvements in process of care for monitoring and treatment of asthma and chronic obstructive pulmonary disease (COPD) in primary care [41].

### Diagnostic assistants

Three studies tested diagnostic assistants [15,35,45]--these CCDSSs provided advice for the diagnosis of acute health conditions, such as acute small bowel obstruction in surgical inpatients [15]. All studies assessed process of care outcomes with two (67%) showing improvements with the CCDSS [35,45]. Roukema *et al. *tested a CCDSS that provided advice for the diagnostic management of children with fever without an apparent source in the emergency department (ED) and showed an increase in test ordering [35]. Stengel *et al. *examined a CCDSS that assisted electronic documentation of diagnosis and findings in patients admitted to orthopaedic wards [45]. In comparison to standard paper forms, the CCDSS demonstrated success in improving diagnosis per patient.

Of the two studies examining patient outcomes, neither demonstrated an improvement.

### Medication dosing assistants

Fourteen studies evaluated medication dosing assistants, providing recommendations specific to drug dosing adjustments, such as insulin dosing or dosing advice for warfarin initiation [14,16-18,21,22,24,27,28,30,32,36,49,50]. These CCDSSs showed improvements in process of care outcomes in 9 of 14 studies (64%) [14,21,22,27,28,32,36,49,50], improvements in patient outcomes in 3 of 10 studies (30%) [32,49,50], and a negative effect on patient outcomes in 1 of 10 (10%) studies [49]. Many studies in the Medication Dosing Assistants section overlap with studies in the therapeutic drug monitoring and thus, are not the primary focus of this review. A more in-depth analysis of these studies is provided in the therapeutic drug monitoring and dosing review (submitted to IS for consideration of publication as part of this series of six reviews).

### Costs and practical process related outcomes

Additional file 5, Table S5 provides data on CCDSS costs and practical process related outcomes, such as the impact on workflow and practitioner satisfaction. Only 11% (4/36) of studies assessed CCDSS monetary costs [16,26,39,40], and 17% assessed other practical process-related outcomes [33,34,39,45,47,49].

## Discussion

Our systematic review identified 36 RCTs of CCDSSs for acute care. The trials were diverse in CCDSS design, clinical settings, clinical problems, and measured effects. Study quality scores increased over time, but that may be due to an improvement in the reporting of trials. Most studies evaluated process of care effects, with 63% showing benefit (benefit was based on at least 50% of the relevant study outcomes being statistically significantly positive) [14,19,21-23,26-28,31,32,34-40,42,45,47-50]. Few examined meaningful patient outcomes, and none showed significant reductions in major patient morbidity or mortality, although some found small reductions in length of hospital stay [16,26,32,40,50]. Lack of findings for patient-important outcomes may be largely an issue of study design, especially the size of the study. Most studies involved few participants, suggesting that they were preliminary in nature, attempting to establish whether the CCDSS could change the process of care, as a prelude to larger studies assessing whether lives could be saved.

Some studies did demonstrate substantial effects on the process of care. For example, the multicentre trial by Paul *et al. *used a causal probabilistic network and local susceptibility data to develop a CCDSS called the TREAT system, which suggested empiric antibiotic regimens based on basic data entered by practitioners during the work-up of patients with new infections [40]. When compared to pathogens isolated further in the course of disease, the TREAT recommendations significantly bettered the physician-only prescriptions (odds ratio of 1.48). Costs were also shown to be reduced, and length of stay was reduced by one day.

In the case of reminders and alerts, where the effect on the process of care is a strong surrogate for patient outcomes, it could also be argued that evidence of effect on patient outcomes is not needed. For example, Terrell *et al. *developed a group of alerts within a computerized physician order entry system to warn emergency physicians when they were about to prescribe potentially dangerous medications (as defined by externally validated criteria) to elderly patients on discharge from the department [48]. The system carried a message explaining why the medication could be dangerous, who had made the recommendation, and links to further explanatory information. Perhaps most importantly, it also suggested safer substitute therapies for each warning. Physicians in the computer-assisted group prescribed fewer inappropriate medications than physicians with no access to the alerts.

It is difficult to make general recommendations regarding the broad applicability and effectiveness of CCDSSs in acute care settings given the current literature and heterogeneity of the individual studies. Several practical details identified as pertinent for extraction by the decision-makers (such as implementation details and costs) were not reported in sufficient detail or with adequate consistency across studies to summarize. There are certainly encouraging trends witnessed by a number of recent high-quality studies demonstrating positive process of care outcomes. Important effects on patient outcomes have yet to be convincingly demonstrated, however, and purchasers and decision makers are advised to take this into consideration.

We did not complete a formal analysis of the factors associated with success across the trials. However, confirming findings from our previous review [3], when study authors were also the developers of the CCDSSS under assessment, the findings were more likely to be favourable for the CCDSS. This could be due to any number of factors including, for example, greater attention to customization for local settings, choosing outcomes more likely to be influenced by CCDSSs, influence of developers on enthusiasm for use of the system, or bias in the analysis of findings, which has been documented for commercial trials of pharmaceuticals [51]. As such, the included trials are potentially more likely to overestimate the size of the effect and increase the risk of a type I error in this review.

### Limitations

A number of studies did not report relevant data or had insufficient data to conduct the appropriate analyses. For example, we were unable to evaluate the effect size for process of care or patient outcomes for several of the studies. As well, our strict inclusion criteria that included only RCTs focuses on only the most scientifically sound studies and would miss, for example, more effective CCDSSSs that were not as rigorously tested. The heterogeneity between studies in CCDSS features and outcome measures was too great to justify a meta-analysis to pool effect sizes. Instead, the overall results of the trials were reported by taking the number of trials with statistically significant results and dividing them by the total number of trials, a method known as vote-counting, which is limited by giving equal weight to each study, regardless of individual merit and size. A study was considered to have a positive effect (*i.e.*, CCDSS showed improvement) if at least 50% of the relevant study outcomes were statistically significantly positive. Many of the studies were small, increasing the risk of type 2 (false negative) error. On the other hand, it is likely that publication bias exists in this field, as shown in many others, such that the number of 'negative' trials is underestimated from the published literature. The majority of studies were conducted in the US and academic medical institutions, where the nature of the clinical landscape could have affected the application and results from the CCDSSs, reducing their generalizability to other settings. Last, but perhaps most important, very few studies evaluated patient-important outcomes.

### Future research directions

Future research should focus on evaluating the effect of CCDSSs on patient outcomes, providing full details of the CCDSS to help establish the relationship between CCDSS characteristics and CCDSS success, and ensuring studies of high methodological quality. Fortunately, with recent initiatives on the adoption of electronic medical records to achieve meaningful enhancements of healthcare [52], there are many opportunities for good measurement and assessment of CCDSSs in acute care. The high quality studies to date show that it is feasible to rigorously evaluate CCDSSs; the findings so far underscore that existing CCDSSs have not been shown to improve patient-important outcomes.

## Conclusions

The majority of CCDSSs demonstrated improvements in process of care but patient outcomes were less likely to be evaluated and far less likely to show positive results. CCDSSs for acute medical care have not matured to degree that clinical decision makers should embrace the technology for clinical application.

## Competing interests

RBH, NLW, JAM, LWK, TN, JCP, NS, RL, and AR received support through the Canadian Institutes of Health Research Synthesis Grant: Knowledge Translation KRS 91791 for the submitted work. RBH is acquainted with several CCDSS developers and researchers, including authors of papers included in this review.

## Authors' contributions

RBH was responsible for study conception and design; acquisition, analysis, and interpretation of data; drafting and critical revision of the manuscript; obtaining funding; study supervision. He is the guarantor. NS acquired, analyzed, and interpreted the data; and drafted the manuscript. RL analyzed and interpreted data; and critically revised the manuscript. AR drafted the manuscript. JAM acquired, analyzed, and interpreted data; drafted the manuscript; critically revised the manuscript; and provided administrative, technical, or material support. JCP acquired, analyzed, and interpreted data; drafted the manuscript; provided statistical analysis; and provided administrative, technical, or material support. LWK and TN acquired data and drafted the manuscript. NLW acquired, analyzed, and interpreted data; drafted the manuscript; provided administrative, technical, or material support; and provided study supervision. All authors read and approved the final manuscript.

## Supplementary Material

Additional file 1 **Study methods scores for trials of acute care management**. Methods scores for the included studies. Click here for file

Additional file 2 **CCDSS characteristics for trials of acute care management**. CCDSS characteristics of the included studies. Click here for file

Additional file 3 **Study characteristics for trials of acute care management**. Study characteristics of the included studies. Click here for file

Additional file 4 **Results for CCDSS trials of acute care management**. Details results of the included studies. Click here for file

Additional file 5 **Costs and CCDSS process-related outcomes for trials of acute care management**. Cost and CCDSS process-related outcomes for the included studies. Click here for file
