# Supplementary material for: Computerized clinical decision support systems for acute care management: A decision-maker-researcher partnership systematic review of effects on process of care and patient outcomes
Source: Implement Sci. 2011 Aug 3;6:91. doi: 10.1186/1748-5908-6-91 (PMC3169487; doi:10.1186/1748-5908-6-91)
Supplement: Additional file 1 — Study methods scores for trials of acute care management. Methods scores for the included studies. [file 1748-5908-6-91-S1.DOCX]

**Additional file 1, Table S1. Study methods scores for trials of acute care management ^a^**

| **Study** | **Allocation concealed ^b^** | **Cluster randomization** | **Adjustment for baseline differences** | **Objective outcome** | **Adequate follow-up** | **Total score** |
| --- | --- | --- | --- | --- | --- | --- |
| Rodman, 1984[27] | 2 | 0 | 2 | 2 | 0 | 6 |
| White, 1984[31] | 0 | 0 | 2 | 2 | 0 | 4 |
| Hurley, 1986[24] | 2 | 0 | 2 | 2 | 2 | 8 |
| Carter, 1987[17] | 0 | 0 | 0 | 2 | 0 | 2 |
| White, 1987[32] | 0 | 0 | 2 | 2 | 2 | 6 |
| Begg, 1989[14] | 0 | 0 | 2 | 2 | 0 | 4 |
| Gonzalez, 1989[22] | 0 | 0 | 2 | 2 | 1 | 5 |
| Hickling, 1989[21] | 0 | 0 | 0 | 2 | 1 | 3 |
| Wyatt, 1989[33] | 2 | 0 | 0 | 2 | 1 | 5 |
| Burton, 1991[16] | 0 | 2 | 2 | 2 | 0 | 6 |
| Casner, 1993[18] | 0 | 0 | 1 | 2 | 0 | 3 |
| Hales, 1995[20] | 0 | 0 | 2 | 2 | 0 | 4 |
| Overhage, 1996[25] | 2 | 2 | 2 | 2 | 2 | 10 |
| Overhage, 1997[26] | 2 | 2 | 2 | 2 | 0 | 8 |
| Vadher, 1997[30] | 0 | 0 | 2 | 2 | 2 | 6 |
| Poller, 1998[28] | 0 | 0 | 0 | 2 | 1 | 3 |
| Kuperman, 1999[23] | 2 | 0 | 0 | 2 | 0 | 4 |
| Dexter, 2001[19] | 2 | 2 | 2 | 2 | 2 | 10 |
| Bogusevicius, 2002[15] | 2 | 0 | 2 | 1 | 2 | 7 |
| Selker, 2002[29] | 2 | 0 | 2 | 2 | 2 | 8 |
| Zanetti, 2003[47] | 2 | 0 | 2 | 2 | 2 | 8 |
| Brothers, 2004[46] | 2 | 0 | 2 | 2 | 0 | 6 |
| Hamilton, 2004[44] | 2 | 0 | 2 | 2 | 2 | 8 |
| Stengel, 2004[45] | 2 | 0 | 2 | 2 | 2 | 8 |
| Rood, 2005[34] | 2 | 0 | 2 | 2 | 2 | 8 |
| Kroth, 2006[39] | 2 | 1 | 0 | 2 | 2 | 7 |
| Kuilboer, 2006[41] | 2 | 2 | 2 | 2 | 2 | 10 |
| Paul, 2006[40] | 2 | 2 | 2 | 2 | 2 | 10 |
| Davis, 2007[42] | 2 | 1 | 2 | 2 | 2 | 9 |
| Peterson, 2007[36] | 0 | 0 | 0 | 2 | 2 | 4 |
| Rothschild, 2007[37, 38] | 2 | 1 | 0 | 2 | 2 | 7 |
| Helder, 2008[43] | 0 | 0 | 2 | 2 | 2 | 6 |
| Roukema, 2008[35] | 0 | 0 | 2 | 2 | 2 | 6 |
| Saager, 2008[50] | 0 | 0 | 2 | 2 | 2 | 6 |
| Cavalcanti, 2009[49] | 2 | 0 | 2 | 2 | 2 | 8 |
| Terrell, 2009[48] | 2 | 1 | 2 | 2 | 2 | 9 |

^a^ Based on five individual items (score 2 = yes, 1 = partly, and 0 = no) and a summed total score (range 0 to 10). Because this review update included only randomized, controlled trials, the total score differs from that reported in the previous version of this review[3]: the item evaluating study type (randomized, quasi-randomized, or concurrent controls) has been replaced by one that evaluates use of concealed allocation (concealed, unclear, not concealed).

^b^If allocation concealment was not readily apparent from the description provided in the published article the primary author of the trial confirmed or indicated that allocation was concealed
