# Supplementary material for: Computerized clinical decision support systems for acute care management: A decision-maker-researcher partnership systematic review of effects on process of care and patient outcomes
Source: Implement Sci. 2011 Aug 3;6:91. doi: 10.1186/1748-5908-6-91 (PMC3169487; doi:10.1186/1748-5908-6-91)
Supplement: Additional file 2 — CCDSS characteristics for trials of acute care management. CCDSS characteristics of the included studies. [file 1748-5908-6-91-S2.DOCX]

**Additional file 2, Table S2. CCDSS characteristics for trials of acute care management** ^a^

| **Study** | **Design** | | | **Interface description** | | | | | **Data entry source** | | | | | | **Methods for delivery of recommendations** | | | | | | | **CCDSS users** | | | | | | **Other characteristics** | | | | |
| --- | --- | --- | --- | --- | --- | --- | --- | --- | --- | --- | --- | --- | --- | --- | --- | --- | --- | --- | --- | --- | --- | --- | --- | --- | --- | --- | --- | --- | --- | --- | --- | --- |
|  | **Stand Alone** | **Integrated with EMR** | **Integrated with CPOE** | Graphic user interface | User must type | Drop down menus | Drag and drop | Other interface | **Automated through EMR** | **Project staff** | **Existing staff** | **Practitioner/decision-maker** | **Patient** | **Other data entry** | Desktop/Laptop computer | E-Mail | PDA | Pager | Project staff | Existing non-prescribing staff | Other Methods | **Trainees** | **Physicians** | **Advanced Practice Nurses** | **Physician Assistants** | **Pharmacists** | **Other health professionals** | Pilot tested | Users trained | Feedback at time of care | CCDSS suggested diagnoses/ treatments/procedures | Authors as developers |
| Rodman, 1984[27] | **+** | **-** | **-** | ? | ? | ? | ? | ? | **-** | **-** | **-** | **+** | **-** | **-** | - | - | - | - | - | + | - | **-** | **+** | **-** | **-** | **+** | **-** | + | + | + | + | + |
| White, 1984[31] | **-** | **+** | **-** | ? | ? | ? | ? | ? | **+** | **-** | **-** | **-** | **-** | **-** | - | - | - | - | - | + | - | **+** | **+** | **-** | **-** | **-** | **-** | - | + | + | + | + |
| Hurley, 1986[24] | **+** | **-** | **-** | - | ? | ? | ? | ? | **-** | **-** | **+** | **-** | **-** | **-** | - | - | - | - | + | - | - | **-** | **+** | **-** | **-** | **-** | **-** | + | - | ~ | + | - |
| Carter, 1987[17] | **+** | **-** | **-** | ? | ? | ? | ? | ? | **-** | **-** | **+** | **-** | **-** | **-** | - | - | - | - | + | - | - | **+** | **+** | **-** | **-** | **-** | **-** | + | + | ~ | + | - |
| White, 1987[32] | **+** | **-** | **-** | ? | ? | ? | ? | ? | **-** | **-** | **+** | **+** | **-** | **-** | + | - | - | - | - | - | - | **+** | **+** | **-** | **-** | **+** | **-** | - | + | + | + | + |
| Begg, 1989[14] | **+** | **-** | **-** | ? | ? | ? | ? | ? | **-** | **-** | **-** | **+** | **-** | **-** | ? | ? | ? | ? | ? | ? | ? | **-** | **+** | **-** | **-** | **-** | **-** | - | - | + | + | + |
| Gonzalez, 1989[22] | **+** | **-** | **-** | ? | ? | ? | ? | ? | **?** | **?** | **?** | **?** | **?** | **?** | ? | ? | ? | ? | ? | ? | ? | **+** | **+** | **-** | **-** | **-** | **-** | ? | + | + | + | - |
| Hickling, 1989[21] | **+** | **-** | **-** | ? | ? | ? | ? | ? | **?** | **?** | **?** | **?** | **?** | **?** | ? | ? | ? | ? | ? | ? | ? | **-** | **+** | **-** | **-** | **-** | **-** | - | - | + | + | + |
| Wyatt, 1989[33] | **+** | **-** | **-** | - | + | + | - | - | **-** | **-** | **-** | **-** | **-** | **+** | - | - | - | - | - | + | - | **+** | **-** | **-** | **-** | **-** | **+** | + | + | + | + | + |
| Burton, 1991[16] | **+** | **-** | **-** | ? | ? | ? | ? | ? | **?** | **?** | **?** | **?** | **?** | **?** | ? | ? | ? | ? | ? | ? | ? | **+** | **+** | **-** | **-** | **-** | **-** | - | - | + | + | - |
| Casner, 1993[18] | **+** | **-** | **-** | ? | ? | ? | ? | ? | **-** | **-** | **-** | **+** | **-** | **-** | - | - | - | - | + | - | - | **-** | **+** | **-** | **-** | **-** | **-** | - | + | + | + | - |
| Hales, 1995[20] | **-** | **+** | **-** | ? | ? | ? | ? | ? | **-** | **-** | **+** | **-** | **-** | **-** | - | - | - | - | - | + | - | **-** | **+** | **-** | **-** | **-** | **+** | - | - | - | - | - |
| Overhage, 1996[25] | **-** | **+** | **+** | ? | ? | ? | ? | ? | **+** | **-** | **-** | **-** | **-** | **-** | + | - | - | - | - | - | + | **+** | **+** | **-** | **-** | **-** | **-** | - | - | + | + | + |
| Overhage, 1997[26] | **-** | **+** | **+** | ? | ? | ? | ? | ? | **+** | **-** | **-** | **+** | **-** | **-** | + | - | - | - | - | - | - | **+** | **+** | **-** | **-** | **-** | **-** | - | - | + | + | + |
| Vadher, 1997[30] | **+** | **-** | **-** | ? | ? | ? | ? | ? | **-** | **+** | **-** | **-** | **-** | **-** | - | - | - | - | + | - | - | **-** | **-** | **+** | **-** | **-** | **-** | + | - | + | + | + |
| Poller, 1998[28] | **+** | **-** | **-** | ? | ? | ? | ? | ? | **-** | **-** | **-** | **+** | **-** | **-** | + | - | - | - | - | - | - | **-** | **+** | **-** | **-** | **-** | **-** | + | + | + | + | - |
| Kuperman, 1999[23] | **-** | **+** | **+** | - | + | ? | ? | ? | **+** | **-** | **-** | **-** | **-** | **-** | + | - | - | - | - | - | - | **+** | **-** | **-** | **-** | **-** | **-** | - | + | + | + | + |
| Dexter, 2001[19] | **-** | **+** | **-** | ? | ? | ? | ? | ? | **+** | **+** | **+** | **-** | **-** | **-** | + | - | - | - | - | - | - | **+** | **+** | **-** | **-** | **-** | **-** | + | - | + | + | + |
|  |  |  |  |  |  |  |  |  |  |  |  |  |  |  |  |  |  |  |  |  |  |  |  |  |  |  |  |  |  |  |  |  |
| Bogusevicius, 2002[19] | **+** | **-** | **-** | ? | ? | ? | ? | ? | **?** | **?** | **?** | **?** | **?** | **?** | ? | ? | ? | ? | ? | ? | ? | **-** | **+** | **-** | **-** | **-** | **-** | + | - | + | + | + |
| Selker, 2002[29] | **+** | **-** | **-** | ? | ? | ? | ? | ? | **-** | **-** | **+** | **-** | **-** | **-** | - | - | - | - | - | + | - | **-** | **+** | **-** | **-** | **-** | **-** | + | - | + | + | - |
| Zanetti, 2003[47] | **-** | **+** | **?** | ? | ? | ? | ? | + | **+** | **-** | **+** | **-** | **-** | **-** | + | - | - | - | - | + | - | **-** | **+** | **+** | **-** | **-** | **-** | ? | ? | + | + | + |
| Brothers, 2004[46] | **+** | **-** | **-** | ? | ? | ? | ? | ? | **-** | **-** | **-** | **+** | **+** | **-** | - | + | - | - | - | - | - | **-** | **+** | **+** | **-** | **-** | **-** | + | + | + | + | + |
| Hamilton, 2004[44] | **?** | **?** | **?** | ? | ? | ? | ? | ? | **-** | **+** | **-** | **-** | **-** | **-** | ? | ? | ? | ? | ? | ? | ? | **-** | **-** | **-** | **-** | **-** | **+** | ? | ? | + | ? | ? |
| Stengel, 2004[45] | **+** | **-** | **-** | + | + | + | - | + | **-** | **+** | **-** | **+** | **-** | **-** | - | - | + | - | - | - | - | **+** | **+** | **-** | **-** | **-** | **-** | + | + | + | + | + |
| Rood, 2005[34] | **-** | **+** | **?** | + | + | ? | ? | + | **+** | **-** | **-** | **-** | **-** | **-** | + | - | - | - | - | - | - | **-** | **+** | **-** | **-** | **-** | **+** | + | + | + | + | + |
| Kroth, 2006[39] | **-** | **+** | **?** | + | + | + | - | ? | **-** | **-** | **-** | **+** | **-** | **-** | + | - | - | - | - | - | - | **+** | **-** | **+** | **-** | **-** | **+** | + | + | + | + | + |
| Kuilboer, 2006[41] | **-** | **+** | **-** | ? | ? | + | ? | + | **+** | **-** | **-** | **-** | **-** | **-** | + | - | - | - | - | - | - | **-** | **+** | **-** | **-** | **-** | **-** | + | + | + | + | + |
| Paul, 2006[40] | **+** | **-** | **-** | ? | - | - | - | + | **-** | **+** | **-** | **-** | **-** | **-** | + | - | - | - | - | - | - | **-** | **+** | **-** | **-** | **-** | **-** | + | - | + | + | + |
| Davis, 2007[42] | **-** | **+** | **+** | ? | ? | ? | ? | + | **+** | **-** | **-** | **-** | **-** | **+** | + | - | + | - | - | - | - | **+** | **+** | **-** | **-** | **-** | **+** | + | + | + | + | + |
| Peterson, 2007[36] | **-** | **-** | **+** | ? | + | + | - | ? | **-** | **-** | **-** | **+** | **-** | **-** | + | - | - | - | - | - | - | **+** | **+** | **+** | **-** | **+** | **-** | + | + | + | + | + |
| Rothschild, 2007[37, 38] | **-** | **+** | **+** | + | + | - | - | + | **-** | **-** | **-** | **+** | **-** | **-** | + | - | - | - | - | - | - | **+** | **+** | **-** | **-** | **-** | **-** | + | - | + | + | + |
| Helder, 2008[43] | **?** | **?** | **?** | ? | ? | ? | ? | ? | **-** | **+** | **-** | **-** | **-** | **-** | + | - | - | - | - | - | - | **+** | **-** | **+** | **-** | **-** | **-** | ? | ? | + | + | ? |
| Roukema, 2008[35] | **+** | **-** | **-** | + | + | + | ? | ? | **-** | **-** | **+** | **-** | **-** | **-** | + | - | - | - | - | - | - | **-** | **+** | **+** | **-** | **-** | **-** | ? | + | + | + | + |
| Saager, 2008[50] | **+** | **-** | **-** | ? | ? | ? | ? | ? | **?** | **?** | **?** | **?** | **?** | **?** | ? | ? | ? | ? | ? | ? | ? | **-** | **-** | **-** | **-** | **-** | **+** | ? | ? | + | + | ? |
| Cavalcanti, | **+** | **-** | **-** | + | ? | ? | ? | ? | **-** | **-** | **-** | **-** | **-** | **+** | + | - | + | - | - | - | - | **-** | **-** | **-** | **-** | **-** | **+** | + | + | + | + | + |
| 2009[49] |  |  |  |  |  |  |  |  |  |  |  |  |  |  |  |  |  |  |  |  |  |  |  |  |  |  |  |  |  |  |  |  |
| Terrell, 2009[48] | **-** | **+** | **+** | + | + | - | - | + | **-** | **-** | **-** | **+** | **-** | **-** | + | - | - | - | - | - | - | **+** | **+** | **-** | **-** | **-** | **-** | - | - | + | + | + |

Abbreviations: CCDSS, computerized clinical decision support system; CPOE, computerized physician order entry system; EMR, electronic medical record; PDA, personal digital assistant. ^a^Symbol key: +, characteristic present; -, characteristic absent; ~, characteristic sometimes present; ?, unstated or uncertain.
