# Supplementary material for: Computerized clinical decision support systems for acute care management: A decision-maker-researcher partnership systematic review of effects on process of care and patient outcomes
Source: Implement Sci. 2011 Aug 3;6:91. doi: 10.1186/1748-5908-6-91 (PMC3169487; doi:10.1186/1748-5908-6-91)
Supplement: Additional file 3 — Study characteristics for trials of acute care management. Study characteristics of the included studies. [file 1748-5908-6-91-S3.DOCX]

**Additional file 3, Table S3. Study characteristics for trials of acute care management^a^**

| **Study (Country)** | **Methods Score^b^** | **Funding Source** | **Indication** | **No. of Practitioners / Patients** | **Setting^c^**  **(No. of clinics / sites)** | **CCDSS Intervention** | **Comparison** |
| --- | --- | --- | --- | --- | --- | --- | --- |
| **Management Assistants – Alerts and Reminders** | | | | | | | |
| Terrell, 2009[48], USA | 9 | Public | Reduction of prescription of potentially inappropriate medications to older adults discharged from emergency departments. | 63 / 5162 | •Academic centre •Emergency Department (1/1) | CCDSS data was only provided when a physician in the intervention group attempted to prescribe 1 of the 9 targeted potentially inappropriate medications in patients ≥ 65 years old who were being discharged from the emergency department. The system provides either an option to order a recommended alternative therapy or to reject the recommendation. When the latter option was chosen, a second menu was displayed to query the most important reason for rejecting the CCDSS recommendation. | Usual care |
| Peterson, 2007[36]^d^, USA | 4 | Private | Drug dosing for patients ≥ 65 years in a tertiary care academic health centre. | 778 / 2981 | •Academic centre  (1/...) | CCDSS provided initial dose advice for sedatives, neuroleptics, anti-emetics, and skeletal muscle relaxants and discouraged prescription of contraindicated drugs for patients ≥ 65 years old in emergency rooms, intensive care units, and subacute units. Practitioners were not prevented from selecting higher doses than recommended. | Control group not specified (probably usual care with no CCDSS recommendations) |
| Kroth, 2006[39], USA | 7 | Public | Improve accuracy of temperature capture by nurses at the bedside of non-critical care hospital patients. | 337 / … | •Other •Academic centre •Hospital inpatients  (.../...) | CCDSS identified patients’ low temperature values and generated prompts for nurses to repeat the measurement. Nurses could take or override the recommendation. | Usual care |
| Rood, 2005[34], The Netherlands | 8 | ... | Management of glucose regulation in critically ill inpatients. | 104 / 484 | •Other •Hospital inpatients (1/1) | CCDSS monitored the interval between glucose measurements and made guideline-based recommendations for timing between glucose measurements and administration of insulin doses in ICU patients. Recommendations were displayed electronically in pop-up windows when patient records were activated. | Paper-based guideline for timing between glucose measurements and administration of insulin doses in ICU patients |
| Zanetti, 2003[47], USA | 8 | Public | Redosing of prophylactic antibiotics during prolonged cardiac surgery. | ... / 447 | •Academic centre •Hospital inpatients (1/1) | CCDSS provided an automated audible alarm and visual intraoperative alert on the operating room computer console for physicians to redose prophylactic antibiotics during cardiac surgery at 225 minutes after administration of preoperative antibiotics. A reply was required to clear the display. If planned redosing was indicated, a new alarm and alert were issued after 30 minutes and the circulating nurse was required to indicate whether a follow-up dose of antibiotics had been administered. | Usual care |
| Selker, 2002[29], USA | 8 | Public | Management of thrombolytic and overall reperfusion therapy in acute myocardial infarction. | ... / 1596 | •Academic centre •Hospital inpatients •Primary care  (28/28) | Thrombolytic Predictive Instrument (TPI) is an electrocardiograph-based CCDSS. When there is an ST segment elevation on the electrocardiogram (ECG), TPI prints on ECG text header its prediction of five key outcomes of thrombolytic therapy for acute myocardial infarction patients. | Usual care |
| Dexter, 2001[19], USA | 10 | Public | Preventive therapies in hospital inpatients. | 202 / 3416 | •Academic centre •Hospital inpatients  (.../...) | CCDSS provided guideline-based reminders for preventative care procedures (pneumococcal vaccination, influenza vaccination, prophylactic entericoated aspirin for cardiovascular disease, and prophylactic subcutaneous heparin for thromboembolic events) to physicians and medical students. | Usual care |
| Kuperman, 1999[23], USA | 4 | Public | Detection of critical laboratory results in hospital inpatients. | ... / ... | •Academic centre •Hospital inpatients (1/1) | The CCDSS was used to detect critical laboratory results for all medical and surgical inpatients and page the health provider that the results were ready. The intervention signalled single laboratory results, changes in laboratory results and detection of drug-laboratory interaction. | Usual care |
| Overhage, 1997[26], USA | 8 | Public | Identification of corollary orders to prevent errors of omission for tests and treatments in hospital inpatients on a general medicine ward. | 92 / 2181 | •Academic centre •Hospital inpatients (1/1) | A rule-based reminder CCDSS determined corollary orders for 87 target orders and displayed these on-line to physicians using the CPOE system. Corollary orders could be accepted or rejected by physicians. | Physicians used CPOE system but did not receive on-line corollary orders. |
| Overhage, 1996[25], USA | 10 | Public | Compliance with 22 US Preventive Services Task Force preventive care measures for hospital inpatients, including cancer screening, preventive screening and medications, diabetes care reminders, and vaccinations. | 78 / 1622 | •Academic centre •Hospital inpatients (1/1) | CCDSS was incorporated into the electronic medical record and order-entry system and used data from these sources to generate reminders for 22 preventive care measures. CCDSS ran overnight and provided reminders to physicians in 2 ways: printed at the top of daily patient reports, and displayed at the bottom of the workstation screen in red when physicians entered orders for patients. Physicians could accept or reject orders generated by the reminder program. | Usual care |
| White, 1984[31], USA | 4 | ... | Monitoring signs and risk factors for digoxin intoxication in inpatients. | ... / 396 | •Academic centre •Hospital inpatients (1/1) | CCDSS (Health Evaluation through Logical Processing [HELP]) accessed a clinical patient database nightly and used expert-determined decision criteria to identify concerns (drug interactions or signs of potential digoxin intoxication) for patients taking digoxin. Concerns were summarized in alert reports placed in patient charts. | Usual care |
| **Management Assistants – Guidelines and Algorithms** | | | | | | | |
| Helder, 2008[43], The Netherlands | 6 | ... | Management of incubator settings in neonatal ICU. | 117 / 136 | •Other •Academic centre •Hospital inpatients (1/1) | CCDSS used infant birth weight, gestational and postnatal ages, room air temperature, incubator design, and use of phototherapy to suggest incubator air temperature and humidity levels for premature, low birth weight neonates. | Nurse-determined incubator humidity levels |
| Davis, 2007[42], USA | 9 | Public | Electronic prescribing for paediatric care (conditions included acute otitis media, allergic rhinitis, sinusitis, constipation, pharyngitis, croup, urticaria, and bronchiolitis) in outpatient and primary care settings. | 44 / 12195 | •Hospital outpatients •Primary care (2/2) | Physicians used an electronic prescription writer on 1 of several computer work stations or wireless hand-held computers to prescribe antibiotics (including selection of indication and treatment duration). CCDSS then displayed evidence-based data relating to the prescription. Full articles or article abstracts were available if requested. | Usual care |
| Rothschild, 2007[37, 38], USA | 7 | Public | Decision support for non-emergent inpatient transfusion orders. | 1414 / 3903 | •Academic centre •Hospital inpatients (1/1) | CCDSS suggested new orders if blood products (red blood cells, platelets, and fresh frozen plasma) ordered through CPOE were inconsistent with guidelines. Recommendations could be overridden. | Usual care |
| Kuilboer, 2006[41], The Netherlands | 10 | Public | Monitoring and treatment of asthma and COPD in daily practice in primary care. | 40 / 156772 | •Primary care  •Solo practice (32/32) | CCDSS uses data in electronic health records and clinical guidelines to provide feedback on treatment to physicians for patients with asthma or COPD. | Usual care |
| Paul, 2006[40], Italy, Germany & Israel | 10 | Public | Management of antibiotic treatment in hospital inpatients. | ... / 2326 | •Academic centre •Hospital inpatients (15/3) | By imputing variables that significantly influence the probability of pathogens, physicians used the TREAT CCDSS to assess the probability of infection, pathogen distribution, mortality and antibiotic coverage, and prescribe empirical antibiotic treatment for microbiologically documented infections. | In control wards only, observation and data collection was conducted and physicians could not access the CCDSS. |
| Brothers, 2004[46], USA | 6 | Public | Surgical management of patients with peripheral arterial disease. | 3 / 206 | •Academic centre  •Hospital outpatients  •Subspecialty clinic  (2/2) | Markov surgical decision analysis CCDSS predicted quality-adjusted life years for each of 4 therapeutic interventions and recommended optimal treatments. Analysis was based on patient data (e.g., utility assessment) and surgeon data (e.g., surgeon surgical results). | Standard decision-making |
| Hamilton, 2004[44], Canada & USA | 8 | ... | Evaluating labour progress and need for caesarean sections. | ... / 4993 | •Academic centre •Hospital inpatients •Hospital outpatients (7/7) | CCDSS used data from clinical examination and obstetrical monitor to create a reference range of women in the same labour conditions. System assigned a percentile ranking of the labour progress of that particular mother against the reference population. This information was used by physicians to determine whether to deliver the baby by caesarean section. | Labour progress was evaluated by plotting cervical dilation against time. |
| Hales, 1995[20], USA | 4 | ... | Computer system for hospital admission screening. | ... / 1971 | •Other •Academic centre (1/1) | A personal CCDSS (Review Criteria) used data from the (Health Evaluation through Logical Processing, [HELP]) hospital information system and data input by nurses to prescreen patients and identify unnecessary hospital admissions. Nurses consulted with physicians about unnecessary admissions. Physicians had the final decision. | Nurses had access to expert system output but did not contact physicians about admissions identified as unnecessary. |
| Wyatt, 1989[33], UK | 5 | Public | Identification of high-cardiac risk patients among patients with chest pain attending the emergency department. | 15 / 153 | •Academic centre •Hospital outpatients •Emergency Department (1/1) | CCDSS, (Admitted to the CCU OR Not, [ACORN]), used questionnaire data manually entered by a nurse to identify the cardiac risk of emergency department patients with chest pain. For those patients identified as high-risk, a nurse took the record to the doctor for urgent consideration. | A questionnaire with 24 questions was administered but data were not entered into CCDSS. |
| **Diagnostic Assistants** | | | | | | | |
| Roukema, 2008[35], The Netherlands | 6 | Public | Diagnostic management for children with fever without apparent source in emergency department. | 15 / 164 | •Emergency Department (1/1) | CCDSS used prediction rules to generate a serious bacterial infection risk score for children < 17 years of age presenting to the emergency department with a fever without apparent source. For patients with high-risk: Users of CCDSS were given advice to “order laboratory tests” for patients randomized to CCDSS intervention. | Same as intervention except that users of CCDSS were not given “order laboratory tests” instruction for children in control group. (This is not explicitly stated in article.) |
| Stengel, 2004[45], Germany | 8 | ... | Diagnosis in patients admitted to orthopaedic ward. Purpose of study was to compare thoroughness of documentation of clinical findings. | 6 / 78 | •Academic centre •Hospital inpatients •Hospital outpatients •Subspecialty clinic  (1/1) | Handheld CCDSS guides entry of patient signs and symptoms and offers clinically reasonable diagnoses for physician selection in orthopaedic hospital ward. Data were transferred to desktop unit daily. | Usual, handwritten, documentation on standard paper forms |
| Bogusevicius, 2002[15], Lithuania | 7 | ... | Diagnosis of acute small bowel obstruction in surgical inpatients. | ... / 80 | •Academic centre •Hospital inpatients (1/1) | CCDSS used a Bayesian posterior probability formula and 36 significant historical, clinical, and laboratory test results together with plain abdominal radiography to diagnose type of mechanical acute small bowel obstruction (complete or partial). Physicians determined appropriate treatment based on diagnosis. | Contrast radiography used for diagnosis |
| **Medication Dosing Assistants** | | | | | | | |
| Cavalcanti, 2009[49], Brazil | 8 | Public, Private | Glucose measurement and insulin dosing for glucose control for ICU patients | 60 / 168 | •Academic centre •Hospital inpatients (5/5) | CCDSS (Computer Assisted Insulin Protocol, [CAIP]) used patient data including current infusion rate, glucose level and time between previous glucose measurements to make recommendations for intravenous insulin dosing and glucose monitoring to maintain a blood glucose between 100 and 130 mg/dL. The CCDSS was available via desktop or handheld computers for nursing staff at hospital based ICUs. The nurses input patient data and followed the recommendations provided. Recommendations were determined by the authors who created the algorithms. | Leuven: A strict glycaemic control protocol for intravenous insulin infusion with target blood glucose between 80 and 110 mg/dL. All insulin adjustments were made by nurses. Conventional: Subcutaneous insulin was administered for blood glucose levels > 150 mg/dL according to a sliding scale. All insulin adjustments were made by nurses. |
| Saager, 2008[50], USA | 6 | Public | Glucose management in diabetic patients in cardiothoracic ICUs. | ... / 40 | •Academic centre •Hospital inpatients (1/1) | CCDSS (EndoTool Glucose Management System) recommended insulin dose, glucose determination frequency, and a 50% dextrose dose (when appropriate) for hypoglycaemia, based on blood glucose readings from a point-of-care device. It used the previous 4 dose responses to regulate the dosing relationship, and is designed to be used by trained health care professionals. | Standard locally-developed paper-based ICU insulin protocol with target blood sugar levels between 90 and 150 mg/dL |
| Peterson, 2007[36]^d^, USA | 4 | Private | Drug dosing for patients ≥ 65 years of age in a tertiary care academic health centre. | 778 / 2981 | •Academic centre  (1/...) | CCDSS provided initial dose advice for sedatives, neuroleptics, anti-emetics, and skeletal muscle relaxants and discouraged prescription of contraindicated drugs for patients ≥ 65 years old in emergency rooms, intensive care units, and subacute units. Practitioners were not prevented from selecting higher doses than recommended. | Control group not specified (probably usual care with no CCDSS recommendations) |
| Poller, 1998[28], UK, Denmark, Portugal, Norway | 3 | Public | Anticoagulation therapy initiation and maintenance for outpatients. | ... / 285 | •Academic centre •Hospital outpatients (5/5) | CCDSS (DAWN AC) generated anticoagulant dosing schedules and time to next INR test using 2 main modules. The induction module was for dosing initial warfarin therapy over the first 4 days to reach a dose within 1 mg of eventual maintenance dose. The maintenance module adjusted the dose to reach and sustain the therapeutic range. | Usual care |
| Vadher, 1997[30], UK | 6 | Public | Warfarin initiation and maintenance for inpatients and outpatients with deep vein thrombosis, pulmonary embolus or systemic embolus, atrial fibrillation, valve disease, or mural thrombus, or who needed prophylaxis. | 49 / 148 | •Academic centre •Hospital inpatients (1/1) | CCDSS used simple proportional-derivative control methods to provide recommendations for initial and maintenance dosing of oral anticoagulation. Maintenance dosing was based on previous dose and difference between target and actual INR. Physicians could choose to accept or reject dosing recommendations, and also received guidelines on anticoagulation. | Usual care plus guidelines for anticoagulation |
| Casner, 1993[18], USA | 3 | ... | Theophylline dosing for inpatients with asthma or COPD. | ... / 47 | •Hospital inpatients (1/1) | Pharmacokinetic CCDSS (linear one-compartment model) was used to predict theophylline infusion rates to achieve a target serum level of 15 mg/L. The CCDSS was run on hand-held computers and adjusted dosing based on 2 early measures of serum theophylline levels. | Physician-directed theophylline infusion adjustments to achieve a target serum level of 15 mg/L based on 2 early serum measures |
| Burton, 1991[16], USA | 6 | Public | Aminoglycoside dosing for inpatients with clinical infections. | ... / 147 | •Academic centre •Hospital inpatients (1/1) | CCDSS with Bayesian-based algorithm used serum aminoglycoside level data to predict aminoglycoside dosage needed to achieve peak (gentamicin and tobramycin, 5-10 mg/L; amikacin, 20-30 mg/L) and trough (gentamicin and tobramicin, <2mg/L; amikacin, <5mg/L) target levels. | Physician-directed aminoglycoside dosing using serum level data |
| Begg, 1989[14], New Zealand | 4 | ... | Individualized aminoglycoside dosing for inpatients receiving gentamicin or tobramycin. | ... / 50 | •Hospital inpatients (.../...) | CCDSS used pharmacokinetic analysis (one-compartment model) to predict individualized aminoglycoside doses and dose intervals needed to achieve a peak level at end of infusion of 8 mg/L and trough level of 1.5 mg/L. | Routine clinical practice used to achieve peak plasma aminoglycoside levels of 6-10 mg/L and trough levels of 1-2 mg/L |
| Gonzalez, 1989[22], USA | 5 | Private | Drug-dosing of aminophylline in the emergency department. | ... / 67 | •Academic centre •Hospital inpatients (.../...) | CCDSS used Bayesian pharmacokinetic model to estimate aminophylline loading and maintenance dosing for individual patients to achieve serum theophylline levels of 15 mg/L (12 mg/L if oral theophylline given within 6 hours). | Dosing nomogram based on emergency department asthma guidelines and theophylline level 4 hours after initial bolus were used to determine aminophylline dosing needed to maintain a serum theophylline level of 10-20 mg/L |
| Hickling, 1989[21], New Zealand | 3 | Private | Pharmacokinetic dosage prediction for aminoglycosides based on estimated creatinine clearance in critically ill patients. | ... / 32 | •Hospital inpatients (1/1) | CCDSS pharmacokinetic model was used to predict early therapeutic dose and dose interval of aminoglycoside to achieve any desired peak and trough concentration in critically ill patients, based on 3 post-distributional plasma concentrations after the initial dose. | Nomogram was used to determine the aminoglycoside dose and dose interval based on estimated creatinine clearance. |
| Carter, 1987[17] USA | 2 | Public | Warfarin initiation dosing for hospital inpatients. | ... / 54 | •Academic centre •Hospital inpatients (1/1) | CCDSS suggested warfarin dosages (analog-computer method) or a single dosage prediction was made using a formula (linear-regression method) for adult inpatients. | Usual care |
| White, 1987[32], USA | 6 | ... | Warfarin initiation and dosing for patients hospitalized with deep vein thrombosis, cerebrovascular accident, transient ischemic attack, pulmonary embolus or atrial fibrillation. | ... / 75 | •Academic centre •Hospital inpatients  (2/...) | CCDSS (Warfcalc) used Bayesian forecasting methods to determine appropriate warfarin dosing based on patient data including response to warfarin therapy. Warfarin therapy was managed by a physician or pharmacist familiar with the CCDSS but who were not experts in management of warfarin therapy. Primary physicians selected target prothrombin ratio. | Usual care. Physicians selected target prothrombin ratio. |
| Hurley, 1986[24], Australia | 8 | Public, Private | Theophylline dosing for inpatients with acute air-flow obstruction. | ... / 96 | •Hospital inpatients (1/1) | Initial loading and infusion doses of theophylline were based on a nomogram; subsequent infusion and oral doses were adjusted based on CCDSS pharmacokinetic analysis of theophylline serum levels. | Physicians selected IV and oral doses of theophylline based on drug serum levels without use of formal pharmacokinetic analysis. |
| Rodman, 1984[27], USA | 6 | Public | Lidocaine dosing for patients in ICUs or coronary care units. | ... / 20 | •Academic centre •Hospital inpatients (1/1) | CCDSS recommended lidocaine infusion regimen based on patient’s age, sex, height, weight, cardiac index, past lidocaine therapy, and desired lidocaine concentration for ICU and coronary care unit patients. | Usual care |

Abbreviations: CCDSS, computerized clinical decision support system; COPD, chronic obstructive pulmonary disease; CPOE, computerized physician order entry system; ICU, intensive care unit; INR, international normalized ratio.

^a^ Ellipses (…) indicate item was not assessed.

^b^ Based on 5 individual items (score 2 = yes, 1 = partly, and 0 = no) and a summed total score (range 0 to 10). Because this review update included only randomized, controlled trials, the total score differs from that reported in the previous version of this review[3]: the item evaluating study type (randomized, quasi-randomized, or concurrent controls) has been replaced by one that evaluates use of concealed allocation (concealed, unclear, not concealed).

^c^ Diabetes clinic is an example of a subspecialty clinic.

^d^Study included in 2 categories.
