# Supplementary material for: Computerized clinical decision support systems for acute care management: A decision-maker-researcher partnership systematic review of effects on process of care and patient outcomes
Source: Implement Sci. 2011 Aug 3;6:91. doi: 10.1186/1748-5908-6-91 (PMC3169487; doi:10.1186/1748-5908-6-91)
Supplement: Additional file 4 — Results for CCDSS trials of acute care management. Details results of the included studies. [file 1748-5908-6-91-S4.DOCX]

**Additional file 4, Table S4. Results for CCDSS trials of acute care management**

| **Study** | **Process of care outcomes** | **CCDSS vs. control data** | **Patient outcomes** | **CCDSS vs. control data** | **CCDSS process of care effect ^a^** | **CCDSS patient effect ^a^** |
| --- | --- | --- | --- | --- | --- | --- |
| **Management Assistants – Alerts and Reminders** | | | | | | |
| Terrell, 2009[48] | **Primary**  **1. Number (%) of ED visits by older adults that resulted in prescriptions for 1 or more of the 9 targeted inappropriate medications; OR (95% CI), *P*-value.**  Prespecified secondary  2. Number (%) of all prescribed medications that were potentially inappropriate; OR (95% CI), *P*-value.  Prespecified  3. Number of times that each potentially inappropriate medication was initially prescribed (n)/ changed to an alternate treatment (n, %) in the CCDSS group vs. prescribed in the control group (n).  3a. Promethazine  3b.Diphenhydramine  3c. Diazepam  3d. Propoxyphene with acetaminophen  3e. Hydroxyzine  3f. Amitriptyline  3g. Cyclobenzaprine  3h. Clonidine  3i. Indomethacin  3j. All inappropriate medications | 1. 69 (2.6%) vs. 99 (3.9%); 0.55 (0.34 to 0.89), *P*= .02  2. 69 (3.4%) vs. 103 (5.4%); 0.59 (0.41 to 0.85), *P*=.006  3a. 32 / 19 (59%) vs. 40  3b. 22 / 8 (36%) vs. 15  3c. 18 / 5 (28%) vs. 10  3d. 8 / 2 (25%) vs. 9  3e. 15 / 6 (40%) vs. 9  3f. 1 / 0 (0%) vs. 8  3g. 5 / 2 (40%) vs. 7  3h. 3 / 2 (67%) vs. 4  3i. 10 / 5 (50%) vs. 1  3j. 114 / 49 (43%) vs. 103 | ... | … | + | … |
| Peterson, 2007[36]^b^ | **1. Median (IQR) ratio of overall prescribed to recommended doses (primary)**  2. Median (IQR) ratio of prescribed to recommended doses by type (not prespecified)  2a. antihistamine/anti-emetic  2b. benzodiazepines  2c. neuroleptics  2d. antihypertensives  2e. NSAIDs  2f. antispasmodics  2g. opiates  2h. sulfonylureas  2i. other anticholinergic  2j. other  2k. beers criteria medications  2l. scheduled  2m. PRN  2n. single dose  2o. multiple dose  2p. non-critical care unit  2q. critical care unit and procedure suites  2r. emergency room  2s. subacute unit  3. median (IQR) ratio of overall prescribed to recommended doses by physicians in the intervention group only vs. physicians in the control group only (not prespecified)  4. percentage of recommended doses selected (not prespecified) | 1. 2.5 (1.0,4.0) vs. 3.0 (1.5, 5.0) (*P<*.001) 2a. 4.0 [2.0, 4.0] vs. 4.0 [2.0, 6.0] 2b. 2.0 [1.0, 4.0] vs. 2.5 [1.2, 4.2] 2c. 4.0 [1.0, 10] vs. 4.0 [1.0, 10] 2d. 2.0 [1.0, 4.0] vs. 2.0 [1.0, 4.0] 2e. 4.0 [1.5, 4.0] vs. 4.0 [2.0, 4.0] 2f. 2.0 [1.0, 4.0] vs. 3.0 [1.1, 6.0] 2g. 1.0 [0.5, 1.5] vs. 1.0 [0.4 , 1.5] 2h. 4.0 [2.0, 6.5] vs. 4.0 [2.0, 8.0] 2i. 2.5 [2.0, 5.0] vs. 2.5 [1.0, 5.0] 2j. 1.0 [1.0, 1.6] vs. 1.3 [1.0, 2.0] 2k. 2.0 [1.0, 4.0] vs. 2.0 [1.0, 4.0] 2l. 2.0 [1.0, 4.0] vs. 2.0 [1.0, 4.0] 2m. 4.0 [3.0, 6.0] vs. 4.0 [3.0, 7.5] 2n. 1.0 [1.0, 2.0] vs. 1.25 [1.0, 2.0] 2o. 4.0 [2.0, 6.0] vs. 4.0 [2.0, 6.0] 2p. 2.5 [1.0, 4.0] vs. 3.0 [1.3, 5.0] 2q. 3.0 [1.5, 6.0] vs. 3.0 [2.0, 6.0] 2r. 2.0 [1.0, 4.0] vs. 2.0 [1.0, 4.0] 2s. 3.0 [1.5, 6.0] vs. 4.0 [2.0, 4.0]  3. 2.0 [1.0,4.0] vs. 4.0 [2.0,6.0] (*P<*.001) 4. 28.6% vs. 24.1% (*P<*.001) | ... | … | + | … |
| Kroth, 2006[39] | **1. Proportion of low temperatures recorded by nursing personnel type (registered nurse / licensed practical nurse / nursing aide/ nursing student/ total) (primary)**  Not prespecified  2. Proportion of temperatures recorded by group (intervention vs. control) within temperature window (degrees F)  2a. < 80  2b. 80 to 90  2c. 90.1 to 95.0  2d. 95.1 to 96.4  2e. 96.5 to 98  2f. 98.1 to 99.0  2g. 99.1 to 100  2h. 100.1 to 102  2i. 102.1 to 104  2j. 104.1 to 106  2k. 106.1 to 110  2l. > 110  2m. 97.0 to 101.5  2n. < 95 or > 110  2o. < 96.4  3. Number of low body temperatures collected by each group on first attempt.  4. Most frequently stored temperature (number)  5. Average temperature recorded.  6. Number of instances of low temperature measurement per patient | 1. 1.9%/1.9%/3.0%/2.7%/2.8% vs. 5.9%/5.0%/5.6%/7.3%/5.7%, *P*<.001  2a. 0.02% vs. 0.02% 2b. 0.01% vs. 0.07% 2c. 0.46% vs. 1.14% 2d. 2.28% vs. 4.45% 2e. 32.20% vs. 28.19% 2f. 37.44% vs. 37.20% 2g. 18.03% vs. 18.88% 2h. 8.50% vs. 8.92% 2i. 0.97% vs. 1.01% 2j. 0.03% vs. 0.05% 2k. 0% vs. 0% 2l. 0.05% vs. 0.07% 2m. 91.23% vs. 88.71% 2n. 0.05% vs. 1.3% 2o. 2.8% vs. 5.7%  3. 2451 vs. 2516  4. 98.4’F (3214) vs. 98.4’F (3158)  5. 97.7’% vs. 96.4’F 6. 7.8 vs. 14.5 | ... | … | + | … |
| Rood, 2005[34] | **1. Deviation between advised and actual glucose measurement times over 10 weeks; (prespecified); N for samples 2352 vs. 2597**  **1a. For late measurements: Mean minutes (SD); proportion of time (SD); difference in proportion of time (95% CI).**  **1b. For early measurements: proportion of time (SD); difference in proportion of time (95% CI).**  **2. Proportion of time that patients’ glucose levels were within specified range over 10 wks (observed difference; 95% CI) (prespecified)**  **2a, Target range, 4.0 to 7.0 mmol/L.**  2b. <2.5 mmol/L  2c. 2.5 to 4 mmol/L  2d. 7 to 8.5 mmol/L  2e. >8.5 mmol/L  3. Proportion of dosing recommendations followed over 10 weeks (observed difference; 95% CI) (not prespecified)  **4. % adherence to guideline for timing of glucose measurement over 10 weeks (observed difference; 95% CI) (prespecified).**  **4a. % samples taken on time.**  4b. % samples taken too late.  4c. % samples taken too early  Pre- and post-intervention periods are available in article. | 1a. 27.95 (118.3) vs. 42.49 (139.5); 28.1% (103.3) vs. 41.9% (99.1); 14% (11 to 16)  1b. 27.8% (28.8) vs. 28.95% (29.3)  2. Total time (minutes): 272,939 vs. 318,403  2a. 54.2% vs. 52.9% (1.3%, 1.0 to 1.56) 2b. 0.09% vs. 0.05% (difference NR) 2c. 1.28% vs. 1.32% (difference NR) 2d. 26.64% vs. 27.53% (difference NR) 2e. 17.79% vs. 18.21% (diff NR)  3. 77.3% vs. 64.2% (13,1%, 11 to 16): total N of samples: 2352 vs. 2597  4. Total N of samples: 2352 vs. 2597 4a. 40.18% vs. 35.54% (4.6%, 2.0 to 7.4) 4b. 25.51% vs. 31% (5.5%, 3.0 to 8.0) 4c. 34.31% vs. 33.46% (difference NR)  Other details regarding pre- and post-intervention periods available. | ... | … | + | … |
| Zanetti, 2003[47] | **1. Number (proportion) of patients given an intraoperative redose of antibiotics, n (%); adjusted OR (95% CI). (primary outcome)** | 1. 93/137 (68%) vs. 55/136 (40%); 3.31 (1.97 to 5.61), *P*<.001.  Note: 227 vs. 222 randomized; 168 vs. 163 could have reminders activated (i.e. surgery documented as >225 mins and patient given antibiotics); and 137 vs. 136 were documented as eligible for intraoperative redosing according to guidelines and were included in primary analysis. | **1. Number (proportion) with surgical-site infection. (secondary outcome)** | 1. 5/137 (4%) vs. 8 /136 (6%); *P*=.4 | + | 0 |
| Selker, 2002[29] | **No clearly prespecified outcomes – subgroup analyses not prespecified.**  **1a. Number of patients who had ST-segment elevation detected but did not have AMI.**  **1b. Number (%) of patients in 1a who received thrombolytic therapy**  **1c. Number (%) of patients who received thrombolytic therapy and had contraindications**  **2. The effect of the CCDSS (TPI) on treatment of patients with AMI: % of patients, RR (95% CI) (adjusted), P-value**  2a. all patients; thrombolytic therapy within 1 hour  2b. all patients; thrombolytic therapy  **2c. all patients, thrombolytic therapy or PTCA**  2d. patients with inferior AMI; thrombolytic therapy within 1 hour  2e. patients with inferior AMI; thrombolytic therapy  2f. patients with inferior AMI, thrombolytic therapy or PTCA  2g. patients with anterior AMI; thrombolytic therapy within 1 hour  2h. patients with anterior AMI; thrombolytic therapy  2i. patients with anterior AMI, thrombolytic therapy or PTCA  3. The effect of the CCDSS (TPI) on treatment of patients with AMI: % of patients, RR (95% CI) (adjusted), P-value  3a. women; thrombolytic therapy within 1 hour  3b. women; thrombolytic therapy  3c. women; thrombolytic therapy or PTCA  3d. men; thrombolytic therapy within 1 hour  3e. men; thrombolytic therapy  3f. men; thrombolytic therapy or PTCA  4. The effect of the CCDSS (TPI) on treatment of patients with AMI for whom physician consultation was entirely by telephone: % of patients, RR (95% CI) (adjusted), P-value  4a. thrombolytic therapy within 1 hour  4b. thrombolytic therapy  4c. thrombolytic therapy or PTCA  5. The effect of the CCDSS (TPI) on treatment of patients with acute myocardial infarction who presented to hospitals without an on-site emergency department physician: % of patients, RR (95% CI) (adjusted), P-value  5a. thrombolytic therapy within 1 hour  5b. thrombolytic therapy  5c. thrombolytic therapy or PTCA | 1a. 208 vs. 191 1b. 3 (1.4%) vs. 1 (0.5%), *P*>.2 1c. 1 (0.3%) vs. 2 (0.6%), *P*>.2  2a. 53.3% vs. 52.5%, 1.0 (0.9 to 1.2), *P*>.2 2b. 62.1% vs. 60.5%, 1.1 (0.96 to 1.1), *P*=.2 2c. 70.3% vs. 67.6%, 1.0 (0.97 to 1.1), *P*=.2 2d. 58.6% vs. 53.2%, 1.1 (0.9 to 1.3), *P*=.08 2e. 67.6% vs. 61.1%, 1.1 (1.01 to 1.2), *P*=.03 2f. 74.7% vs. 67.7%, 1.1 (1.01 to 1.2), *P*=.03 2g. 45.3% vs. 51.4%, 0.9 (0.8 to 1.1), *P*>.2 2h. 53.9% vs. 59.5%, 0.9 (0.8 to 1.1), *P*>.2 2i. 63.8% vs. 67.6%, 1.0 (0.8 to 1.1), *P*>.2  3a. 48.4% vs. 40.5%, 1.2 (0.96 to 1.5), *P*=.10 3b. 58.2% vs. 48.1%, 1.2 (1.01 to 1.5), *P*=.03 3c. 65.7% vs. 55.7%, 1.2 (1.0 to 1.4), *P*=.04 3d. 55.9% vs. 58.0%, 1.0 (0.9 to 1.1), *P*>.2 3e. 64.2% vs. 66.2%, 1.0 (0.9 to 1.1), *P*>.2 3f. 72.8% vs. 73.1%, 1.0 (0.9 to 1.1), *P*>.2  4a. 53.6% vs. 41.1%, 1.3 (1.01 to 1.7), *P*=.04 4b. 63.2% vs. 47.3%, 1.3 (1.2 to 3.1), *P*=.01 4c. 66.4% vs. 50.7%, 1.3 (1.1 to 1.6), *P*=.01  5a. 58.8% vs. 40.9%, 1.4 (0.8 to 2.6), *P*=.19 5b. 76.5% vs. 50.0%, 1.5 (0.97 to 2.4), *P*=.04 5c. 79.4% vs. 54.6%, 1.5 (0.96 to 2.2), *P*=.05 | **1. Proportion of patients who died within 30 day follow-up (*P*-value)**  **2. Number (%) of strokes within 30 day follow-up (*P*-value).**  **3. Number (%) of thrombolysis-related bleeding events that required transfusion during the 30 day follow-up (*P*-value).** | 1. 5.0 vs. 3.4 (*P*=.15) 2. 3 (0.5%) vs. 3 (0.5%) (*P*>.2) 3. 22 (5.8%) vs. 16 (4.5%) (*P*>.2) | 0 | 0 |
| Dexter, 2001[19] | **(primary outcomes-"the rates at which the various preventive therapies were ordered")**  **1. Proportion of hospitalizations with an order for therapy**  **1a. Pneumococcal vaccination**  **1b. Influenza vaccination**  **1c. Prophylactic heparin**  **1d. Prophylactic aspirin at discharge**  **2. Proportion of hospitalizations during which therapy was ordered for an eligible patient**  **2a. Pneumococcal vaccination**  **2b. Influenza vaccination**  **2c. Prophylactic heparin**  **2d. Prophylactic aspirin at discharge** | 1a. 8.5% vs. 0.9%, *P*<.001 1b. 5.4% vs. 0.4%, *P<*.001  1c. 10.5% vs. 8.2%, *P*<.001  1d. 29.7% vs. 25.4%, *P*<.001 2a. 35.8% vs. 0.8%, *P*<.001  2b. 51.4% vs. 1.0%, *P*<.001  2c. 32.2% vs. 18.9%, *P*<.001 2d. 36.4% vs. 27.6%, *P*<.001 | ... | … | + | … |
| Kuperman, 1999[23] | **1. (primary outcome) length of time interval from filing alerting result to ordering of appropriate treatment (in hours) [median (IQR), mean (SD), range, *P*-value] 1a. all**  1b. when alerting situation satisfied laboratory’s critical reporting criteria and a phone call was made  1c. when alerting situation did not satisfy laboratory’s critical reporting criteria  2. (secondary outcome) interval between results filing time and resolution of critical condition (in hours) (for all cases, intervention vs. control cases given in median, mean, range, p value).  2a. all 2b. when alerting situation satisfied the laboratory’s critical reporting criteria and a phone call was made  2c. when alerting situation did not satisfy laboratory’s critical reporting criteria | 1a. 1.0 (0.2-2.6), 4.1 (12.1), 0-100.5 vs. 1.6 (0.6-4.2), 4.6 (9.1), 0.1-66.1 median *P*=.003, mean *P*=.003  1b. 0.7 (0.2-2.6), 3.4 (8.0), 0-44.6) vs. 1.1 (0.6-3.0), 3.3 (7.4), 0.1-55.1, median *P*=.06, mean *P*=.59  1c. 1.2 (0.2-2.9), 4.8 (14.8), 0-100.5 vs. 2.5 (0.9-6.5), 6.1 (10.7), 0.1-66.1, median *P*=.009, mean *P*=.01  2a. 8.4 (4.0-14.5), 14.4 (18.7), 0.2-118.9 vs. 8.9 (5.4-23.2), 20.2 (28.5), 1.3-198.5, median *P*=.11, mean *P*=.11  2b. 7.0 (3.4-14.1), 12.8 (15.4), 0.2-68.1 vs. 8.1 (4.0-18.9), 13.7 (14.5), 1.4-64.7, median *P*=.43, mean *P*=.68  2c. 9.2 (5.6-17.9), 15.8 (21.1), 0.7-118.9 vs. 10.2 (6.8-35.7), 28.8 (38.7), 4.1-198.5, median *P*=.05, mean *P*=.06 | **1. (prespecified) Number (%) of adverse events within 48 hours of alert, (/94 for intervention; /98 for control) *P*-value**  1a. death 1b. cardiopulmonary arrests 1c. an unexpected transfer to the ICU 1d. myocardial infarction  1e. delirium 1f. stroke 1g. new renal insufficiency 1h. new acute renal failure 1i. dialysis 1j. unexpected return to the operating room **1k. all** | 1a. 7 (7.4%) vs. 13 (13.3%), *P*=.19 1b. 2 vs. 1, *P*=.53 1c. 6 vs. 1, *P*=.05 1d. 1 vs. 0, *P*=.3 1e. 4 vs. 3, *P*=.66 1f. 0 vs. 1, *P*=.33 1g. 4 vs. 1, *P*=.16 1h. 1 vs. 1, *P*=.98 1i. 5 vs. 3, *P*=.43 1j. 1 vs. 3, *P*=.33  1k. 31 vs. 27, *P*=.41 | + | 0 |
| Overhage, 1997[26] | Prespecified unless otherwise indicated: **1. % corollary orders with immediate compliance.**  **1a. Overall.**  1b. Excluding saline lock orders (not prespecified).  1c. At 1^st^ order suggestion (not prespecified). **2. % corollary orders with compliance within 24 hours.**  **2a. Overall.**  2b. Excluding saline lock orders (not prespecified).  **3. % corollary orders with compliance during hospital stay.**  **3a. Overall.**  3b. Excluding saline lock orders (not prespecified).  **4. Number of times pharmacists intervened with physicians for significant errors over 6 months.**   5. Compliance with corollary orders within 24 hours for the following 25 most common triggering orders. Total number of orders; % compliance (% increase) (not prespecified).  5a. Heparin infusion  5b. IV fluid orders  5c. cimetidine po  5d. Type and cross.  5e. Insulin lente humulin  5f. Furosemide po  5g. Ferrous sulphate  5h. Furosemide IV  5i. Warfarin.  5j. Ventilator settings.  5k. Insulin Neutral Protamine Hagedorn humulin  5l. Vancomycin IV  5m. Sustained release theophyllin  5n. Gentamicin IV  5o. Insulin reg humulin  5p. Digoxin po  5q. Glyburide po  5r. Meperidine intramuscular/IV  5s. Captopril po  5t. Enteral feeding  5u.Enalapril po  5v.Kayexalate suspension  5w.Timentin IV  5x.Spironolactone po  5y.Glipizide po  6. Compliance with the following 25 most common corollary orders within 24 hours. Total number of orders; % compliance (% increase) (not prespecified).  6a. Serum creatinine  6b. Saline lock  6c. Serum electrolytes  6d. Glycosylated haemoglobin A1.  6e. Activated partial thromboplastin time  6f. Serum glutamic pyruvic transaminase (alanine aminotransferase)  6g. Sodium docusate  6h. Serum glutamic oxaloacetic transaminase (aspartate amintransferase)  6i. Capillary glucose.  6j. Blood cell profile.  6k. Stool occult blood test  6l. Prothrombin time  6m. Theophylline level  6n. Diphenhydramine  6o. Platelet count  6p. Acetominophen  6q. Reticulocyte count  6r. Nasogastric feeding tube  6s. Fe-TIBC  6t. Vancomycin  6u. Phenytoin level  6v. Portable anterior-posterior chest x-ray  6w. arterial-venous blood gas  6x. Simplate bleed time  6y. Gentamicin level | 1a. 46.3% vs. 21.9%, *P*<.001 1b. 46.4% vs. 27.6%, *P*<.001 1c. 48% vs. 23%, *P*<.001  2a. 50.4% vs. 29.0%, *P*<.001 2b. 50.9% vs. 35.3%, *P*<.001 3a. 55.9% vs. 37.1%, *P*<.001  3b. 56.0% vs. 43.5%, *P*<.001 4. 105 vs. 156, *P*=.003  5a. 1476; 77.42% vs. 40.24% (37.18%) 5b. 1061; 64.66% vs. 0%, (64.66%) 5c. 1055; 12.66% vs. 5.18% (7.48%) 5d. 542; 22.90% vs. 14.64% (8.26%) 5e. 518; 40.00% vs. 31.01% (8.99%) 5f. 410; 75.38% vs. 62.09% (13.29%) 5g. 394; 21,43% vs. 16.47% (4.96%) 5h. 360; 60.88% vs. 51.85% (-0.98%) 5i. 303; 68.18% vs. 35.09% (33.09%) 5j. 242; 80.14% vs. 21.78% (58.36%) 5k. 241; 52.17% vs. 26.19% (25.98%) 5l. 224; 60.44% vs. 44.36% (16.08%) 5m. 215; 73.33% vs. 45.46% (27.88%) 5n. 197; 78.35% vs. 61.00% (17.35%) 5o. 197; 53.33% vs. 35.87% (17.46%) 5p. 178; 96.88% vs. 84.15% (12.73%) 5q. 177; 51.28% vs. 43.43% (7.85%) 5r. 177; 24.24% vs. 5.41% (18.84%) 5s. 177; 74.42% vs. 55.06% (19.36%) 5t. 170; 23.08% vs. 7.60% (15.48%) 5u. 161; 73.68% vs. 70.59% (3.10%) 5v. 161; 26.09% vs. 18.48% (7.61%) Article reports difference % as 18.48 (repeat of control group %) – revised to 7.61% - could not confirm with author (no response).  5w. 161; 45.24% s 14.29% (30.95%) 5x. 158; 42.25% vs. 20.69% (21.56%) 5y. 147; 47.22% vs. 36.00% (11.22%)  6a. 1209; 48.28% vs. 41.18% (7.10%) 6b. 1065; 64.73% vs. 0% (64.73%) 6c. 1034; 87.03% vs. 70.86% (16.18%) 6d. 821; 23.71% vs. 7.39% (16.32%) 6e. 615; 89.21% vs. 59.56% (29.65%) 6f. 569; 12.63% vs. 1.87% (10.76%)  6g. 506; 79.35% vs. 79.26% (0.09%) 6h. 467; 7.14% vs. 0% (7.14%) 6i. 446; 30.77% vs. 4.41% (26.36%) 6j. 382; 80.46% vs. 51.44% (29.02%) 6k. 374; 60.94% vs. 12.09% (48.85%) 6l. 320; 64.57% vs. 45.52% (19.05%) 6m. 270; 75.89% vs. 46.51% (29.38%) 6n. 267; 16.41% vs. 7.19% (9.21%) 6o. 236; 70% vs. 15.09% (54.91%) 6p. 232; 19.66% vs. 14.78% (4.88%) 6q. 205; 19.66% vs. 11.36% (8.29%) 6r. 170; 23.08% vs. 7.60% (15.48%) 6s. 149; 12.64% vs. 0% (12.64%) 6t. 143; 90.74% vs. 65.17% (25.57%)  6u. 140; 73.13% vs. 38.36% (34.78%) 6v. 127; 81.69% vs. 33.93% (47.76%) 6w. 123; 72.60% vs. 0% (72.60%) 6x. 123; 26.23% vs. 0% (26.23%) 6y. 118; 90% vs. 75.86% (14.14%) | **Not clearly prespecified**  1. Mean LOHS (days).  2. Maximum serum creatinine level during hospital stay (units NR). | 1. 7.62 vs. 8.12 (difference -0.5, 95% CI -0.17-1.19, *P*=.94) 2. 1.51 (1.25) vs. 1.42 (0.88), *P*=.28 | + | 0 |
| Overhage, 1996[25] | **Primary outcomes**  **1. Compliance with preventive care guidelines over 6 months: No. of eligible patients (% compliance).**  **1a. Overall.**  1b. Cervical cytology study.  1c. Pneumococcal vaccination.  1d. Aspirin.  1e. Oestrogen treatment.  1f. Calcium treatment.  1g. Opthalmologic referral.  1h. Mammography.  1i. Thyroid stimulating hormone screen.  1j. Hepatitis B screen.  1k. Rubella screen.  1l. Screening urinalysis.  1m. Cholesterol test.  1n. Pregnancy test.  1o. Human Immunodeficiency Virus screen.  1p. Angiotensin-converting enzyme inhibitor.  1q. Heparin prophylaxis.  1r. 24 hour urine protein screen.  1s. Sickle cell screen.  1t. Cholesterol treatment.  1u. Screening electrocardiogram.  1v. Beta-blocker.  1w. Sexually transmitted disease screen.  2. Attitude towards providing preventive care to hospitalized patients at 6 months (pre-defined). | 1a. 23% vs. 24%, *P*=.78 1b. 323 (2.8%) vs. 329 (2.8%), *P*=.41  1c. 271 (2.6%) vs. 243 (2.1%), *P*=.69 1d. 246 (9.4%) vs. 247 (9.7%), *P*=.89 1e. 243 (0.8%) vs. 232 0.3%), *P*=.62 1f. 243 (5.4%) vs. 232 (3.9%), *P*=.45 1g. 217 (2.3%) vs. 200 (1.5%), *P*=.55 1h. 125 (5.6%) vs. 131 (1.5%), *P*=.08 1i. 112 (16.1%) vs. 118 (9.3%), *P*=.12 1j. 88 (8.0%) vs. 92 (2.2%), *P*=.08 1k. 80 (1.2%) vs. 86 (0.3%), *P*=.30 1l. 68 (32.4%) vs. 75 (34.7%), *P*=.77 1m. 70 (14.3%) vs. 58 (13.8%), *P*=.94 1n. 60 (13.3%) vs. 66 (13.6%), *P*=.96  1o. 44 (4.6%) vs. 43 (9.3%), *P*=.38 1p. 35 (29.0%) vs. 45 (56.0%), *P*=.02  1q. 30 (43.3%) vs. 28 (35.7%), *P*=.55 1r. 24 (25.0%) vs. 23 (4.4%), *P*=.05  1s. 22 (9.0%) vs. 14 (0%), *P*=.25 1t. 11 (9.1%) vs. 16 (6.2%), *P*=.78 1u. 13 (0%) vs. 14 (21.4%), *P*=.08  1v. 14 (14.3%) vs. 10 (20.0%), *P*=.71  1w. 2 (50%) vs. 6 (16.7%), *P*=.35 2. No difference (data NR) | ... | … | 0 | … |
| White, 1984[31] | **Prespecified**  **1. Number of physician actions related to alerts at 3 months; ratio for alert/nonalert group weighted by number of alerts days (ratio >1 indicates benefit for CCDSS group).**  **1a. Any action.**  1b. Serum digoxin determination ordered.  1c. Digoxin withheld.  1d. Digoxin discontinued.  1e. Digoxin dose reduced.  1f. Quinidine changed.  1g. Beta-blocking agent changed.  1h. Potassium supplement ordered.  1i. Serum potassium determination ordered.  1j. Oxygen delivery increased.  1k. Concern of toxicity in note.  1l. Electrocardiogram ordered.  2. Number of alerts (%) by alert reason for 211 vs. 185 patients (prespecified).  This is descriptive data that is not evaluable for effect.  2a. Any alert.  2b. Low weight.  2c. Old age.  2d. High serum digoxin level.  2e. Low serum potassium level.  2f. Renal insufficiency.  2g. No serum potassium.  2h. Concurrent beta-blocker.  2i. Concurrent quinidine.  2j. Concurrent calcium channel blocker.  2k. Acid-base disorder.  2l. Hypoxemia.  2m. Atrial tachycardia with block.  2n. Junctional arrhythmia.  2o. Ventricula arrhythmia.  2p. Sinoatrial block.  2q. Atrioventricular block.  2r. Acute infarction.  Not prespecified.  3. Number of alert days at 3 months. | 1a. 175 vs. 136 (1.22, *P*<.003) 1b. 48 vs. 17 (2.67, *P*<.001) 1c. 27 vs. 9 (2.84, *P*<.002) 1d. 5 vs. 2 (2.37, *P*<.14) 1e. 5 vs. 1 (4.73, *P*<.06) 1f. 2 vs. 1 (1.89, *P*<.30) 1g. 4 vs. 0 (NR, *P*<.03) 1h. 69 vs. 48 (1.33, *P*<.04) 1i. 117 vs. 89 (1.24, *P*<.02) 1j. 42 vs. 32 (1.24, *P*<.16) 1k. 5 vs. 1 (4.73, *P*<.06) 1l. 36 vs. 29 (1.17, *P* < .25)   2a. 150 (71%) vs. 134 (72%), *P*=NS 2b. 0 (0%) vs. 1 (0.5%), *P*=NS 2c. 8 (4%) vs. 12 (6%), *P*=NS 2d. 8 (4%) vs. 8 (4%), *P*=NS 2e. 21 (10%) vs. 34 (18%), *P* = significant 2f. 15 (7%) vs. 9 (5%), *P*=NS 2g. 2 (1%) vs. 3 (2%), *P*=NS 2h. 19 (9%) vs. 16 (9%), *P*=NS 2i. 12 (6%) vs. 6 (3%), *P*=NS 2j. 2 (1%) vs. 1 (0.5%), *P*=NS 2k. 7 (3%) vs. 4 (2%) , *P*=NS 2l. 45 (20%) vs. 37 (20%), *P*=NS 2m. 1 (0.5%) vs. 0 (0%), *P*=NS 2n. 0 (0%) vs. 2 (1%), *P*=NS 2o. 15 (7%) vs. 10 (5%), *P*=NS 2p. 1 (0.5%) vs. 9 (5%), *P*=NS 2q. 6 (3%) vs. 8 (4%), *P*=NS 2r. 3 (1%) vs. 2 (1%), *P*=NS  3. 260 vs. 246  Note: For 2p (sinoatrial block) - article reports 9 alerts but 0%. Corrected to 5% (9 alerts/185 patients) but could not confirm with author (no response). | ... | … | + | … |
| **Management Assistants – Guidelines and Algorithms** | | | | | | |
| Helder, 2008[43] | **1. Median number (95% CI) of days to regain birthweight (primary)**  2. Mean central body temperature during first 14 days (secondary)  3. Mean incubator temperature (secondary)  4. Mean amount of dexamethasone or indomethacin (secondary)  5. Mean caloric intake (not prespecified)  6. Mean incubator humidity setting (not prespecified) | 1. 9 (8-10) vs. 9 (7-11) 2. not significant 3. results not provided 4. did not differ significantly 5. did not differ significantly 6. did not differ significantly | **1. Proportion with intraventricular  haemorrhage (absent, mild, severe)  2. Proportion of patients with sepsis 3. Number (proportion) of patients who died** | 1. 47%,26%,1% vs. 44%,24%,5% (*P*=.26) 2. 46.5% vs. 38.5% (*P*=.34) 3. 4 (6.2%) vs. 9 (12.7%) (*P*=.20) | 0 | 0 |
| Davis, 2007[42] | **Primary**  **1. Change in proportion of prescriptions consistent with evidence-based recommendations over 18-50 months (adjusted difference, 95% CI).**  By study site: PCC (University of Washington outpatient teaching clinic) or SP (Primary care paediatric clinic) 2. Change in proportion of prescriptions for otitis media consistent with evidence-based recommendations (difference, 95% CI). PCC over 50 months / SP over 18 months 2a. Antibiotic treatment.  2b. Amoxicillin.  2c. Twice daily treatment.  2d. <10 days of antibiotics.  2e. Dosage.  3. Change in proportion of prescriptions for allergic rhinitis consistent with evidence-based recommendations (difference, 95% CI). PCC over 50 months / SP over 18 months 3a. Appropriate treatment choice.  4. Change in proportion of prescriptions for bronchiolitis consistent with evidence-based recommendations at PCC over 50 months (difference, 95% CI). [Insufficient data for SP site] 4a. Albuterol.  5. Change in proportion of prescriptions for sinusitis, pharyngitis, croup, constipation, or urticaria consistent with evidence-based recommendations (difference, 95% CI). PCC over 50 months / SP over 18 months. 5a. Appropriate treatment choice.  Note: Proportional changes were based on individual-prescription-level data; differences were obtained using analyses adjusted for provider clustering and volume of provider visits.  Note: Very limited data were provided for 2 subanalyses: use of a 1-click prescription change option and exploration of provider fatigue over time. | 1. 4% vs. 1% (8%, 1 to 15) 2a. -20% vs. -23% (15%, 2 to 30) / -5% vs. -27% (24%, 8 to 40) 2b. 12% vs. -23% (-2%, -17 to 13) / 3% vs. -7% (12%, -12 to 37) 2c. 20% vs. 36% (-8%, -28 to 11) / 0% vs. 3% (6%, -21 to 32) 2d. 7% vs. 13% (-7%, -21 to 6) / 0% vs. 0% (0%, -0.1 to 0.6) 2e. 7% vs. 15% (9%, -6 to 24) / -10% vs. -3% (-3%, -17 to 11) 3a. 11% vs. 5% (19%, 4 to 35) / 6% vs. -21% (39%, -32 to 110) 4a. 21% vs. 32% (-6%, -18 to 7) 5a. 15% vs. 3% (15%, -1 to 32) / -14% vs. -19% (26%, -41 to 94) | ... | … | + | … |
| Rothschild, 2007[37, 38] | The pre-specified primary outcomes were transfusion guideline adherence of junior house staff at DS intervention (4 months).  1. Appropriateness of transfusion orders. Number (%).  1a. chart review confirms DS-agree (appropriate order)  1b. chart review changes to DS-disagree (inappropriate order)  1c. chart review changes to DS-agree (appropriate order)  1d. chart review confirms DS-disagree (inappropriate order)  **2. Final total appropriateness ratings of DS interventions. Number (%), 2 sided *P-* value**  **2a. Appropriate transfusion decision**  **2b. Inappropriate transfusion decision** | 1a. 305 vs. 349 1b. 106 vs. 121 1c. 108 (11.5%) vs. 154 (14.4%) 1d 698 (74.3%) vs. 922 (85.7%)  2a. 546 (40.4%) vs. 503 (32.5%) *P*<.001 2b. 804 (59.6%) vs. 1043 (67.5%)*P*<.001 | **1. Number of severely undertransfused patients. (primary outcome)** | No evidence of severely undertransfused patients found. | + | 0 |
| Kuilboer, 2006[41] | 1. Median of paired differences of Delta values (the difference between the intervention and baseline periods) (P-value) for each age group: 0-11, 12-39, 40-59, ≥60. (prespecified)  1a. Number of contacts  1b. Number of peak total flow measurements  1c. Number of peak flow ratio measurements  1d. Number of FEV1 total measurements  1e. Number of FEV1 ratio measurements  1f. Number of antihistamines prescriptions  1g. Number of cromoglycate prescriptions  1h. Number of deptropine prescriptions  1i. Number of oral bronchodilators prescriptions  1j. Number of oral corticosteroids prescriptions | 1a. -0.164 (*P*=.26), +0.154 (.03), +0.068 (.76), +0.257 (.13) 1b. +0.020 (.02), +0.029 (.02), +0.028 (.10), +0.005 (.13) 1c. +0.000 (.07), +0.402 (.004), +0.181 (.009), +0.000 (.11) 1d. +0.005 (.03), +0.005 (.06), +0.004 (.009), 0.000 (.11) 1e. +0.000 (.046), +0.056 ((.01), +0.250 (.01), +0.000 (.02) 1f. 0.000 (.88), 0.000 (.50), -0.004 (.08), -0.000 (.32) 1g. 0.000 (.14), -0.0004 (.03), 0.000 (.051), 0.000 (.89) 1h. -0.003 (.75), N/A, N/A, N/A 1i. 0.001 (.81), 0.000 (.66), 0.000 (.12), 0.000 (.23) 1j. -0.004 (.05), -0.002 (.84), -0.023 (.11), -0.045 (.68) | ... | … | 0 | … |
| Paul, 2006[40] | **1. Rate of appropriate antibiotic treatment, intervention intention-to-treat OR (95% CI) *P*-value; intervention per protocol, OR (95% CI) *P*-value per site (n/N(%)) (primary outcome):**  1a. Israel  1b. Germany  1c. Italy  **1d. Overall**  2. Rate of appropriate antibiotic treatment, intervention per protocol, OR (95% CI) p value per site (n/N(%)) (primary outcome):  2a. Israel  2b. Germany  2c. Italy  2d. Overall  3. Number (%) of antibiotics prescribed in Israel / Germany / Italy ): (secondary outcome)  3a. no antibiotic  3b. narrow-spectrum penicillins  3c. piperacillin/tazobactam or sulbactam  3d. first-generation cephalosporin  3e. broad-spectrum cephalosporins  3f. Flouroquinolones  3g. aminoglycosides  3h. glycopeptides  3i. carbapanems | 1a. 140/203 (69.0%) vs. 131/206 (63.6%), 1.27 (0.84 to 1.92) *P*=.25 1b. 38/44 (86.4%) vs. 32/43 (74.4%), 2.18 (0.72 to 6.54) *P*=.16 1c. 38/50 (76.0%) vs. 13/4 (54.2%), 2.68 (0.95 to 7.52) *P*=.06 1d. 216/297 (72.7%) vs. 176/273 (64.5%), 1.48 (1.03 to 2.11) *P*=.03  2a. 74/87 (85.1%) vs. 131/206 (63.6%), 3.26 (1.69 to 6.27) *P*≤.001 2b. 18/19 (94.7%) vs. 32/43 (74.4%), 6.19 (0.74 to 51.91) *P*=.06 2c. 22/28 (78.6%) vs. 13/4 (54.2%), 3.10 (0.93 to 10.39), *P*=.06 2d. 114/134 (85.1%) vs. 176/273 (64.5%), 3.42 (1.97 to 5.96), *P*=.001  3a. 173 (20%) vs. 172 (21%) / 4 (2%) vs. 3 (2%) / 28 (16%) vs. 8 (9%) 3b. 92 (11%) vs. 85 (10%) / 36 (17%) vs. 26 (15%) / 44 (25%) vs. 8 (9%) 3c. 26 (3%) vs. 17 (2%) / 14 (7%) vs. 13 (8%) / 11 (6%) vs. 3 (3%) 3d. 29 (3%) vs. 11 (1%) / 0 vs. 0 / 0 vs. 0 3e. 333 (39%) vs. 405 (49%) / 108 (52%) vs. 84 (49%) / 23 (18%) vs. 37 (43%) 3f. 144 (17%) vs. 98 (12%) / 29 (14%) vs. 29 (17%) / 68 (38%) vs. 28 (32%) 3g. 33 (4%) vs. 15 (2%) / 6 (3%) vs. 8 (5%) / 3 (2%) vs. 1 (1%) 3h. 26 (3%) vs. 21 (3%) / 9 (4%) vs. 8 (5%) / 5 (3%) vs. 6 (7%) 3i. 5 (0.6%) vs. 3 (0.4%) / 9 (4%) vs. 6 (3%) / 6 (3%) vs. 3 (3%) | **1. Mean/median (SD) duration of hospital stay (prespecified)**  1a. Israel  1b. German  1c. Italy  **1d. Overall**  2. Mean/median (SD) duration of hospital stay among patients surviving 30 days (N=1837)  2a. Israel  2b. German  2c. Italy  2d. Overall  **3. Mean/median (SD) duration of fever, median/mean (SD) (pre-specified)**  3a. Israel  3b. German  3c. Italy  **3d. Overall**  **4. Overall 30 day mortality intention to treat, n/N(%)**  4a. Israel  4b. German  4c. Italy  **4d. Overall**  5. Overall 30 day mortality per protocol, n/N(%)  5a. Israel  5b. German  5c. Italy  5d. Overall | 1a. 4/7.21 (9.7) vs. 5/8.04 (11.1), *P*=.01 1b. 10/13.6 (11.2) vs. 14/16.3 (12.0), *P*=.02 1c. 8/12.13 (15.7) vs. 7/11.3 (10.7), *P*=.60 1d. 6/8.83 (11.29) vs. 6/9.45 (11.52), *P*=.06 2a. 4/7.1 (10.2) vs. 5/7.9 (11.6), *P*=.03 2b. 11/16.4 (13.2) vs. 16/19.9 (13.8), *P*=.04 2c. 8/12.2 (15.9) vs. 7/11.4 (10.7), *P*=.59 2d. 5/8.8 (11.9) vs. 5/9.4 (12.2), *P*=.13 3a. 1/2.2 (4.1) vs. 1/2.5 (4.7), *P*=.01 3b. 1/1.9 (2.7) vs. 1/2.1 (3.0), *P*=.49 3c. 3/4.0 (3.4) vs. 3/3.8 (4.3), *P*=.02 3d. 1/2.4 (3.9) vs. 1/2.5 (4.5), *P*=.25 4a. 113/860 (13.1) vs. 128/823 (15.6), *P*=.16 4b. 26/208(12.5) vs. 16/172 (9.3), *P*=.32 4c. 10/177(5.6) vs. 1/86(1.2), *P*=.11 4d. 149/1153(12.9) vs. 145/1012(14.3), *P*=.61 5a. 35/344(10.2) vs. 38/301(12.6), *P*=.33 5b. 9/69(13.0) vs. 6/53(11.3), *P*=.77 5c. 5/120(4.2) vs. 0/42(0), *P*=.33 5d. 49/503(9.7) vs. 44/371(11.9), *P*=.72 | + | 0 |
| Brothers, 2004[46] | **Primary**  **1. Agreement between surgeon’s initial and final treatment plan, % (kappa). Prespecified**  2. Surgeon level of comfort with management decision at 1 week (Provider Decision-Process Instrument, metric NR).  Not clearly prespecified 3. Initial intervention (primary amputation, bypass operation, balloon angioplasty, medical therapy) (number of patients).  4. Intervention within 3 months (primary amputation, bypass operation, balloon angioplasty, medical therapy) (number of patients). 5. Last intervention (primary amputation, bypass operation, balloon angioplasty, medical therapy) (number of patients). | 1. 88% (0.77) vs. 88% (0.81), Not significant 2. 47.2 (4.4) vs. 46.0 (5.1), %), *P*=NS N=100 vs. 106 3. 4,21,6,69 vs. 6,39,5,56, *P*<.1 4. 3,14,5,78 vs. 6,28,3,69, *P*<.1 5. 10,17,4,69 vs. 16,30,5,55, *P*<.1 | … | … | 0 | … |
| Hamilton, 2004[44] | **1. total number (%) of caesarean sections (primary)** 2. total number (%) of vaginal births (not pre-specified)  3. number (%) of pregnancy lengths in each range (not prespecified) 3a. 35-36 weeks 3b. 37-40 weeks 3c. 41 weeks | 1. 436 (17.6%) vs. 425 (16.9%), *P*=.53 2. 2038 (82.3%) vs. 2089 (83.1%), *P*=.53  3a. 107 (4.3%) vs. 99 (3.9%), *P*=.54 3b. 1896 (76.5%) vs. 1981 (78.8%), *P*=.06 3c. 475 (19.2%) vs. 435 (17.3%), *P*=.09 | **1. number (%) of babies with Apgar score in each range 1 minute after birth (secondary) 1a. 0-2 1b. 3-4** **1c. 5-6 1d. 7-8 1e. 9-10 2. number (%) of babies with Apgar score in each range 5 minutes after birth (secondary) 2a. 0-2 2b. 3-4 2c. 5-6 2d. 7-8 2e. 9-10**  **3. rate for the recorded indication of dystocia (pre-specified)**  4. obstetrical and neonatal complications (not prespecified) | 1a. 31 (1.3%) vs. 27 (1.1%), *P*=.65 1b. 63 (2.5%) vs. 55 (2.2%), *P*=.46 1c. 138 (5.6%) vs. 126 (5.0%), *P*=.41 1d. 607 (24.5%) vs. 627 (25.0%), *P*=.74 1e. 1639 (66.2%) vs. 1671 (66.6%), *P*=.83 2a. 7 (0.3%) vs. 8 (0.3%), *P*=.98 2b. 5 (0.2%) vs. 4 (0.2%), *P*=.98 2c. 37 (1.5%) vs. 35 (1.4%), *P*=.85 2d. 186 (7.5%) vs. 201 (8.0%), *P*=.55 2e. 2239 (90.5%) vs. 2261 (90.1%), *P*=.68  3. no data provided  4. 0 vs. 0 | 0 | 0 |
| Hales, 1995[20] | **1. Proportion (number) of hospital admissions considered unnecessary over 6 months.  2. Expected vs. actual % change in unnecessary hospital admissions over 6 months.** | 1. 3.6% (36/992) vs. 3.9% (38/979), *P*>.43  2. 11.6% vs. 6.5%, *P*=NS  Note: Discrepancy in text (Overall Performance, p.730, 11.6% as expected or measured change?). | ... | … | 0 | … |
| Wyatt, 1989[33] | **1. Overall management.**  1a. true positive rate (not prespecified)  1b. false negative rate (prespecified)  1c. false positive rate (not prespecified)  **1d. overall accuracy**  **2. Timings**  2a. Median time (mins, IQR) until patient first saw doctor (not prespecified)  2b. Median time (mins, IQR) in ED (accident and ED) (not prespecified)  **2c. Median time (mins, IQR) until cardiac care unit admission (prespecified)** | 1a. 80% vs. 82% 1b. 20% vs. 18% 1c. 4% vs. 4% 1d. 90% vs. 92%  2a. 37 (17 to 72) vs. 28 (10 to 51) 2b. 103 (72 to 157) vs. 108 (72 to 164) 2c. 118 (84 to 190) vs. 102 (78 to 149) | ... | … | … | … |
| **Diagnostic Assistants** | | | | | | |
| Roukema, 2008[35] | **1.** **Number (proportion) of patients for whom tests were ordered (for intervention group, proportion out of cases in which CCDSS advised to order lab tests)(not clearly pre-specified)** | 1. 61 (82%) vs. 40 (44%) (*P* value not provided but reported as significant) | **1. Median (IQR) time (min) spent at ED (prespecified)** 2. Median (IQR) time (min) spent at ED for patients who had lab tests ordered (not prespecified) | 1. ITT: 138 (104-181) vs. 123 (83 vs. 179) *P*=.16 Per protocol 140 (116-184) vs. 123 (83-179) *P*=.06 2. 149 (116-184) vs. 160 (15-213) *P*=.43 | + | 0 |
| Stengel, 2004[45] | **1a. Median (IQR) number of diagnoses per patient (primary outcome)**  1b. Number (proportion) of ICD codes that were false or redundant  1c. Number of diagnoses per patient after correction for quasi-false-positives  2. Mean (95% CI) coding quality of patient records during the study period (pre-specified secondary outcome)  2a. regularly performed data entry  2b. detailed depiction of clinical findings  2c. correct assessment of patient’s progress and translation into ICD diagnoses  3. Total number of ICD diagnoses generated by each documentation method (not pre-specified) | 1a. 9 (6 to 14) vs. 4 (3 to 5) (*P*<.001) 1b. 48(11.7%) vs. 7 (4.5%); risk difference 7.2%, 95% CI 2.0% to 11.4% 1c. *P*<.001  2a. 1.90 (1.63 to 2.17) vs. 2.71 (2.38 to 3.08) (*P*<.001) 2b. 1.59 (1.38 to 1.86) vs. 2.08 (1.84 to 2.33) (*P*<.005) 2c. 1.87 (1.64 to 2.10) vs. 2.53 (2.34 to 2.83) (*P*<.003)  3. 411 vs. 157 | ... | … | + | … |
| Bogusevicius, 2002[15] | **Prespecified**  **1. Diagnosis of acute SBO (no statistical comparisons)**  **1a. Sensitivity.**  **1b. Specificity.**  1c. Positive predictive value.  1d. Negative predictive value.  **2. Diagnosis of partial SBO (no statistical comparisons)**  **2a. Sensitivity.**  **2b. Specificity.**  2c. Positive predictive value.  2d. Negative predictive value.  **3. Mean (SD) time to diagnosis (hours).**  The Garg paper indicates improvement on practitioner outcomes. Although there is a difference in time to diagnosis, the accuracy data is not compared and the authors conclude that “computer-aided diagnosis had no significant advantage over contrast radiography in the accuracy of diagnosis…”. | 1. Article reports results similar.  1a. 87.5% vs. 76.9% 1b. 100% vs. 100%  1c. 100% vs. 100% 1d. 92.3% vs. 90%  2. Article reports results similar  2a. 100% vs. 100% 2b. 87.5% vs. 76.9% 2c. 92.3% vs. 90% 2d. 100% vs. 100%  3. 1 (NR) vs. 16 (18), *P<*.001 | **Prespecified with follow-up time NR**  **1. Number (proportion) of patients with bowel necrosis.**  **2. Number (proportion) of patients with morbidity.**  **3. Number (proportion) of patient deaths.**  **4. LOHS (days).**  **5. Postoperative LOHS (days).**  **6. Number (proportion) of patients receiving each type of surgical procedure.**  **6a. open lysis of adhesion.**  **6b. laparoscopic lysis of adhesion.**  **6c. bowel resection.**  Note: In Table II, postoperative hospital stay (8 days) was longer than overall hospital stay (6 days). It seems as if these data have been reversed. The author did not respond to a request for clarification. | 1. 1 (3%) vs. 1 (3%), *P*=1.0 2. 4 (10%) vs. 3 (8%), *P*=.76 3. 2 (5%) vs. 0 (0%), *P*=.16 4. 6 vs. 6, *P*=.84 5. 8 vs. 8, *P*=1.0  6a. 17/21 (81%) vs. 10/16 (63%), *P*=.23 6b. 3/21 (14%) vs. 3/16 (19%), *P*=.69 6c. 1/21 (5%) vs. 1/16 (6%), *P*=.90 | 0 | 0 |
| **Medication Dosing Assistants** | | | | | | |
| Cavalcanti, 2009[49] | **All outcomes are presented in the order: CCDSS vs. Leuven vs. Conventional**  **1. Median (IQR) number of BG measurements obtained per patient (secondary)**  **2. Mean (SD) proportion of time with BG controlled between 60 and 140 mg/dL (secondary)** | 1. 100 (33 to 192) vs. 105 (35 to 312) vs. 49(39-77)  *P* [CCDSS vs. Leuven] =.52;  *P* [CCDSS vs. Conventional] =.01  2. 71.8 (18.0) vs. 67.9(20.8) vs. 47.1(30.2);  *P* [CCDSS vs. Leuven] =.50;  *P* [CCDSS vs. Conventional] <.001 | All outcomes are presented in the order: CCDSS vs. Leuven vs. Conventional  **1. Mean of patients’ median BG during the ICU stay (mg/dL) (primary) 2. Number (%) of patients with hypoglycaemia (≥ 1 BG measurement ≤ 40 mg/dL) (primary)** 3. Mean of proportion of patients’ glucose measurements ≤40 mg/dL (secondary) (inconsistency < or ≤40 mg/dL)  4. Median (IQR) hyperglycaemic index, with a cutoff at 140 mg/dL (mg/dL per hour) (secondary) | 1. 125.0 vs. 127.1 vs. 158.5  *P* [CCDSS vs. Leuven] =0.34;  *P* [CCDSS vs. Conventional] <0.001  2. 12 (21.4) vs. 24 (41.4) vs. 2 (3.8); *P* [CCDSS vs. Leuven] =.02;  *P* [CCDSS vs. Conventional] =.006 3. 0.43 vs. 0.55 vs. 0.03 *P* [CCDSS vs. Leuven] =.04;  *P* [CCDSS vs. Conventional] =.007 4. 4.2 (2.0 to 9.6) vs. 8.7 (2.5 to 20.2) vs. 20.5 (5.1 to 42.8);  *P* [CCDSS vs. Leuven] =.10;  *P* [CCDSS vs. Conventional] <.001 | + | + / - |
| Saager, 2008[50] | **Primary outcome=decrease in BG 1 Operating room outcomes:   a) BG in range (90 to 150 mg/dL), %. b) Time in range (minutes)(?mean, SD).   2 ICU outcomes:   a) BG in range (90 to 150 mg/dL), %. b) Time in range (minutes)(?mean, SD).** | 1a) 49% vs. 27%; *P<*.001 1b) 121 (67) vs. 64 (85): *P*=.02  2a) 84% vs. 60%; *P*<.001 2b) 536 (135) vs. 377 (214); *P*=.01 | **Prespecified**  **1 Operating room outcomes:  a) Mean (?SD) BG (BG) (mg/dL).**  **b) Mean (?SD) time to BG<150 mg/dL (min).**  **2 ICU outcomes: a) Mean (?SD) BG (mg/dL).**  **b) Mean (?SD) time to BG<150 mg/dL (min).**  (Outcomes not prespecified)  3. Number of episodes of hypoglycaemia (BG<60 mg/dL). 3a. Operating room. 3b. ICU:  4. Length of surgery, minutes (unclear if mean and SD) 5. Length of cross-clamp, minutes (unclear if mean and SD)  6. Cardiopulmonary bypass times, minutes (unclear if mean and SD) 7. Median ICU length of stay, days (IQR) 8. LOHS, days (IQR) 9. Postoperative complications (arrhythmias, prolonged intubation, infection, stroke or myocardial infarction).  10. Troponin 1, brain natriuteric peptide and ketones, measured at baseline, after removal of cross-clamp, and at 6 and 12 hours after surgery. | 1a) 147 (19) vs. 177 (36); *P<*.001 1b) 62 (92) vs. 91 (121); *P*=.55 2a) 126 (18) vs. 147 (27); *P*=.01 2b) 40 (97) vs. 171 (238); *P*=.02  3a. 1 vs. 0; *P*>0.99  3b. 4 vs. 1; *P* =.60 Note: 3 of 4 episodes of hypoglycaemia in the ICU occurred within the same patient. 4. 290 (67) vs. 281 (82); *P*=.69 5. 85 (34) vs. 77 (29); *P*=.44  6. 135 (33) vs. 123 (43); *P*=.36 7. 2.5 (2 to 6) vs. 2.5 (2 to 4.75); *P*=.83 8. 9.5 (6 to 11.75) vs. 7.0 (6 to 11.75); *P*=.18 9. No differences, data NR 10. No differences at any time point, data NR.  (Author has not responded to multiple queries about results being means and SDs) | + | + |
| Peterson, 2007[36]^b^ | **1. median (IQR) ratio of overall prescribed to recommended doses (primary)**  2. median (IQR) ratio of prescribed to recommended doses by type (not prespecified)  2a. antihistamine/anti-emetic  2b. benzodiazepines  2c. neuroleptics  2d. antihypertensives  2e. NSAIDs  2f. antispasmodics  2g. opiates  2h. sulfonylureas  2i. other anticholinergic  2j. other  2k. beers criteria medications  2l. scheduled  2m. PRN  2n. single dose  2o. multiple dose  2p. non-critical care unit  2q. critical care unit and procedure suites  2r. emergency room  2s. subacute unit  3. median (IQR) ratio of overall prescribed to recommended doses by physicians in the intervention group only vs. physicians in the control group only (not prespecified)  4. percentage of recommended doses selected (not prespecified) | 1. 2.5 (1.0-4.0) vs. 3.0 (1.5- 5.0) (*P<*.001) 2a. 4.0 [2.0-4.0] vs. 4.0 [2.0-6.0] 2b. 2.0 [1.0-4.0] vs. 2.5 [1.2-4.2] 2c. 4.0 [1.0-10] vs. 4.0 [1.0-10] 2d. 2.0 [1.0-4.0] vs. 2.0 [1.0-4.0] 2e. 4.0 [1.5-4.0] vs. 4.0 [2.0-4.0] 2f. 2.0 [1.0-4.0] vs. 3.0 [1.1- 6.0] 2g. 1.0 [0.5-1.5] vs. 1.0 [0.4-1.5] 2h. 4.0 [2.0-6.5] vs. 4.0 [2.0-8.0] 2i. 2.5 [2.0-5.0] vs. 2.5 [1.0-5.0] 2j. 1.0 [1.0-1.6] vs. 1.3 [1.0-2.0] 2k. 2.0 [1.0-4.0] vs. 2.0 [1.0-4.0] 2l. 2.0 [1.0-4.0] vs. 2.0 [1.0-4.0] 2m. 4.0 [3.0-6.0] vs. 4.0 [3.0-7.5] 2n. 1.0 [1.0-2.0] vs. 1.25 [1.0-2.0] 2o. 4.0 [2.0-6.0] vs. 4.0 [2.0-6.0] 2p. 2.5 [1.0-4.0] vs. 3.0 [1.3-5.0] 2q. 3.0 [1.5-6.0] vs. 3.0 [2.0-6.0] 2r. 2.0 [1.0-4.0] vs. 2.0 [1.0-4.0] 2s. 3.0 [1.5-6.0] vs. 4.0 [2.0-4.0]  3. 2.0 [1.0-.0] vs. 4.0[2.0-6.0] (*P<*.001) 4. 28.6% vs. 24.1% (*P<*.001) | ... | … | + | … |
| Poller, 1998[28] | 6-month study with ≥ 3 month follow-up  Data also reported by patient subgroups (below), study weeks (1-3, 4-9, 10-21, >22), and by each of 5 participating centres.  a) Stable on long-term anticoagulant therapy (most >22 weeks therapy)  b) Stabilization group who were discharged from hospital within 6 weeks of starting anticoagulation therapy.  Data also reported by study weeks (1-3, 4-9, 10-21, >22) and the 2 subgroups above.  **Prespecified: proportion of time in range.**  **1. Mean (SD) proportion of time within target INR range for all patients and all ranges (3 ranges used in study: 2-3, 2.5-3.5, and 3-4.5) (days).**  **1a. All patients**  1b. Stabilization patients  1c. Stable patients  2. Stabilization patients – first 3 weeks  2a. Number of INRs.  2b. Proportion of time in target range.  2c. Mean time between visits (days).  2d. Proportion dose changes.  2e. Proportion traditional interventions.  2f. Proportion low INRs.  2g. Proportion high INRs.  2h. Mean INR.  3. Stabilization patients (83 vs. 92 patients) – weeks 4 to >22  3a. Number of INRs.  3b. Proportion of time in target range.  3c. Mean time between visits (days).  3d. Proportion dose changes.  3e. Proportion traditional interventions.  3f. Proportion low INRs.  3g. Proportion high INRs.  3h. Mean (SD) INR.  4. Stable patients (39 vs. 40 patients) – overall  4a. Number of INRs.  4b. Proportion of time in target range.  4c. Mean time between visits (days).  4d. Proportion dose changes.  4e. Proportion traditional interventions.  4f. Proportion low INRs.  4g. Proportion high INRs.  4h. Mean (SD) INR.  5. Total (122 vs. 132 patients)  5a. Number of INRs.  **5b. Proportion of time in target range.**  5c. Mean time between visits (days).  5d. Proportion dose changes.  5e. Proportion traditional interventions.  5f. Proportion low INRs.  5g. Proportion high INRs.  5h. Mean (SD) INR.  6. Proportion low INRs  6a. Stabilization, INR target 2.0 to 3.0.  6b. Stabilization, INR target 2.5 to 3.5.  6c. Stabilization, INR target 3.0 to 4.5.  6d. Stable, INR target 2.0 to 3.0.  6e. Stable, INR target 2.5 to 3.5.  6f. Stable, INR target 3.0 to 4.5.  7. Proportion high INRs  7a. Stabilization, INR target 2.0 to 3.0.  7b. Stabilization, INR target 2.5 to 3.5.  7c. Stabilization, INR target 3.0 to 4.5.  7d. Stable, INR target 2.0 to 3.0.  7e. Stable, INR target 2.5 to 3.5.  7f. Stable, INR target 3.0 to 4.5.  8. Proportion time in INR ranges.  8a. Stable, All ranges  8b. Stable, INR target 2.0 to 3.0.  8c. Stable, INR target 2.5 to 3.5.  8d. Stable, INR target 3.0 to 4.5.  Note: data also reported for stabilization patients by INR target range but this is provided separately by weeks (4-9, 10-21, and >22), not overall. | 1a. 63.3% (28.0) vs. 53.2% (27.7), *P*=.004 1b. 61.8% (27.1) vs. 54.0% (27.5), *P*=.06 1c. 66.4% (29.9) vs. 51.2% (28.4), *P*=.02  2a. 40 vs. 195 2b. 42% vs. 45% 2c. 7 vs. 7  2d. 55% vs. 65% 2e. 35% vs. 0% 2f. 28% vs. 36% 2g. 38% vs. 28% 2h. 3.0 vs. 2.7  3a. 619 vs. 693 3b. 68% vs. 55% 3c. 17 vs. 16 3d. 39% vs. 57% 3e. 23% vs. 0% 3f. 29% vs. 36% 3g. 11% vs. 16% 3h. 2.6 (0.8) vs. 2.6 (1.1)  4a. 314 vs. 387 4b. 72% vs. 59% 4c. 20 vs. 18 4d. 36% vs. 46% 4e. 21% vs. 0% 4f. 25% vs. 27% 4g. 18% vs. 19% 4h. 2.7 (0.9) vs. 2.7 (0.8)  5a. 933 vs. 1080 5b. 70% vs. 56% 5c. 18 vs. 17 5d. 38% vs. 53% 5e. 22% vs. 0% 5f. 28% vs. 33% 5g. 15% vs. 17% 5h. 2.6 (0.9) vs. 2.6 (1.0)  6a. 22.8% vs. 32.2% 6b. 34.5% vs. 44.3% 6c. 35.4% vs. 44.7% 6d. 19.7% vs. 23.0% 6e. 32.2% vs. 23.3% 6f. 42.1% vs. 46.4%  7a. 15.7% vs. 17.7% 7b. 9.1% vs. 19.7% 7c. 9.4% vs. 10.5% 7d. 16.2% vs. 19.4% 7e. 25.3% vs. 18.3% 7f. 5.3% vs. 7.1% 8a. 72.3% vs. 59.3% 8b. 80.0% vs. 59.9% 8c. 51.6% vs. 72.5% 8d. 76.1% vs. 46.3% | ... | … | + | … |
| Vadher, 1997[30] | **Main outcomes**  **1. Median [SE] days to reach therapeutic range (INR ≥2).  2. Median [SE] days to reach stable dose (INR 2-3 for 3 consecutive days).  3. Median time to first pseudoevent (INR ≤1.5 or ≥5 after therapeutic range is reached).**  **For inpatient treatment (n=60 vs. 62)**  **4. Days (per 100 patient days of treatment) at INR 2-3; relative rate (95% CI). (main outcome)**  **For outpatient treatment (n=53 vs. 64) 5. Days (per 100 patient days of treatment) at INR 2-3; (relative rate (95% CI). (main outcome)**  Prespecified For inpatient treatment (n=60 vs. 62) 6. Days (per 100 patient days of treatment) at INR <1.5; relative rate (95% CI). 7. Days (per 100 patient days of treatment) at INR <2.0; relative rate (95% CI). 8. Days (per 100 patient days of treatment) at INR >3.0; relative rate (95% CI)  9. Days (per 100 patient days of treatment) at INR >5.0; relative rate (95% CI).  For outpatient treatment (n=53 vs. 64) 10. Days (per 100 patient days of treatment) at INR <1.5.  11. Days (per 100 patient days of treatment) at INR <2.0; relative rate (95% CI).  12. Days (per 100 patient days of treatment) at INR >3.0; relative rate (95% CI).  13. Days (per 100 patient days of treatment) at INR >5.0; relative rate (95% CI).  Not prespecified  14. Number of patients below therapeutic range at hospital discharge.  15. Number of patients who did not reach a stable dose before study endpoint.  16. Median {range} INR test interval in inpatients (days).  17. Median {range} INR test interval in outpatients (days).  18. Median [SE] days to 1st pseudoevent among inpatients.  19. Number of pseudoevents at median 88-93 days.  20. Number of pseudoevents due to overtreatment. | RRs reported are inverse of those in the paper to be consistent with presentation of data as intervention vs. control.  1. 3 [0.34] vs. 3 [0.29], *P*=.24  2. 7 [0.43] vs. 9 [1.8], *P*=.01  3. Rates NR, *P*=.06  4. 59.4 vs. 52.2; 1.11 (1 to 1.43)  5. 63.7 vs. 51.0; 1.25 (1.11 to 1.42)  6. 1.3 vs. 5.6; 0.24 (0.13 to 0.45)  7. 18.3 vs. 21.4; 0.83 (0.59 to 1.25)  8. 22.3 vs. 26.4; 0.83 (0.59 to 1.25).  9. 1.2 vs. 2.8; 0.42 (0.10 to 1.67)  10. 1.3 vs. 4.2; 0.30 (0.11 to 0.77)  11. 21.1 vs. 31.8; 0.67 (0.48 to 0.91)  12. 15.1 vs. 17.2; 0.91 (0.56 to 1.43)  13. 0.8 vs. 1.1; 0.67 (0.07 to 5)  14. 4/72 vs. 8/76  15. 11/72 vs. 14/76  16. 2 {1 to 22} vs. 2 {1 to 30}, *P*=.07  17. 14 {2 to 63} vs. 14 {1 to 91}, *P*=.2  18. 8.7 [2.32] vs. 7 [2.64], *P*=.03  19. 25 vs. 41  20. 12 vs. 18 | **Prespecified with median follow-up of 88-93 days.**  **1. n/N deaths.**  **2. n/N patients with hemorrhage events.**  **3. n/N patients with thromboembolism events.** | 1. 2/72 vs. 2/76 2. 2/72 vs. 4/76  3. 4/72 vs. 1/76 | 0 | … |
| Casner, 1993[18] | **Prespecified (time NR). 1. Mean serum theophylline levels (mg/L) (SD)**  **1a. ≥ 8 hours after IV therapy had been initiated (C1)**  **1b. ≥ 6 hours after the first measurement (C2)**  **1c. just before discontinuation of the IV theophylline infusion (C3)**  **1d. time interval (mean or median not specified) between C1 and C3 (hours)**  **2. Mean (SD) absolute difference between final and target (15 mg/L) theophylline levels (mg/L).  3. Mean (SD) difference between target (15 mg/L) and mean final theophylline level (mg/L).  4. Number of patients with subtherapeutic (<10 mg/L) final theophylline levels.  5.** **Number of patients with toxic (>20 mg/L) final theophylline levels.**  Not clearly prespecified (no units provided). 6. Mean (SD) pH levels, d1.  7. Mean (SD) pH levels, d2.  8. Mean (SD) pH levels, d3.  9. Mean (SD) PCO2 levels, d1.  10. Mean (SD) PCO2 levels, d2.  11. Mean (SD) PCO2 levels, d3.  12. Mean (SD) clearance (L/hr)  13. Mean (SD) elimination rate constant (hr-1)  14. Mean (SD) half-life (hr)  15. Mean (SD) number of days of theophylline administration  16. Mean (SD) prediction error | 1a. 10.2 (6.4) vs. 9.8 (3.9), *P*=NS 1b. 10.6 (3.3) vs. 9.7 (3.2), *P*=NS 1c. 14.8 (4.4) vs. 12.6 (4.1), *P*=NS 1d. 48 vs. 40, *P*= NS  2. 3.5 (2.7) vs. 3.9 (2.6), *P*=NS  3. 0.21 (4.49) vs. 2.41 (4.07), *P*=NS  4. 4 vs. 3, *P*=NS  5. 1 vs. 1, *P*=NS  6. 7.36 (0.10) vs. 7.36 (0.12), *P*=NS 7. 7.39 (0.08) vs. 7.42 (0.04), *P*= NS 8. 7.39 (0.11) vs. 7.45 (0.07), *P*=NS 9. 43.47 (13.44) vs. 45.19 (13.77), *P*=NS 10. 41.22 (12.04) vs. 36.58 (5.53), *P*=NS 11. 46.50 (14.76) vs. 38.33 (9.42), *P*=NS  12. 6.6 (5.5) vs. 4.2 (2.4), *P*=NS 13. 0.16 (0.09) vs. 0.14 (0.08), *P*=NS 14. 5.3 (2.4) vs. 6.2 (2.9), *P*=NS 15. 4.1 (3.3) vs. 3.2 (1.5), *P*=NS 16. 0.21 (4.49) vs. 2.41 (4.07), *P*>.05 | **Not clearly prespecified.**  **1. Number of patients with theophylline-associated toxicity (nausea, vomiting, tremor, tachycardia, and seizures) (follow-up time NR): n/N 2. Mean (SD) LOHS (days).**  2a. Mean length of hospitalization without one outlier in each group (days)  **3. Mean (SD) duration of treatment (days).** | 1. 1/17 vs. 0/18. Event was tachycardia secondary to high initial theophylline level.  2. 11.4 (21.6) vs. 8.8 (15.4), *P*=NS 2a. 6.1 vs. 5.2, *P*=NS 3. 4.1 (3.3) vs. 3.2 (1.5), *P*=NS | 0 | 0 |
| Burton, 1991[16] | **Not clearly prespecified (follow-up unclear) 1. Mean (SD) beginning aminoglycoside dose (mg/day).  2. Mean (SD) ending aminoglycoside dose (mg/day).  3. Mean (SD) ending aminoglycoside dose interval (h).  4. Mean (SD) peak aminoglycoside level (mg/L).  5. Number (proportion) of patients with peak aminoglycoside level > 4mg/L.  6. Mean (SD) trough aminoglycoside levels (mg/L).  7. Number (proportion) of patients with trough aminoglycoside levels ≥2mg/L.**  **8. Mean (SEM) length of aminoglycoside therapy (days).** | 1. 238 (64.8) vs. 230 (49.7), *P*=NS 2. 272 (92.5) vs. 261 (75.8), *P*=NS 3. 13.0 (3.7) vs. 9.6 (2.9), *P*=NS 4. 5.3 (1.8) vs. 4.4 (1.7), *P*=.001 5. 58/70 (82.9%) vs. 44/73 (60.3%), *P*=NS 6. 1.1 (0.9) vs. 1.2 (0.8), *P*=NS 7. 6/69 (8.7%) vs. 11/75 (14.7%), *P*=NS 8. 7.3 (6.4) vs. 8.3 (0.5), *P*=.09 | **Follow-up time unclear 1. Proportion of patients cured.  2. Proportion of patients with response to therapy.  3. Proportion of patients with treatment failure.  4. Proportion of deaths.  5. Proportion of patients with indeterminate response.  6. Proportion of patients with nephrotoxicity.  7. Mean (SEM) LOHS (days).  8. Mean (SEM) LOHS after start of antibiotics (days).**  8a. sepsis  8b. pneumonia  8c. cellulitis  8d. soft-tissue infections  8e. urinary tract infection  8f. gangrene  8g. postoperative wound infection  8h. peritonitis  8i. neutropenic, empiric therapy  8j. osteomyelitis  8k.cholangitis/cholecystitis  8l. catheter-tip infection  8m. subacute bacterial endocarditis  8n. septic arthritis  8o. pyelonephritis  **8p. overall** | 1. 25.7% vs. 25.3%. *P*=NS 2. 60% vs. 48%, *P*=NS 3. 2.9% vs. 5.3%, *P*=NS 4. 1.4% vs. 4%, *P*=NS 5. 7.1% vs. 8%, *P*=NS 6. 4/72, 5.6% vs. 7/75, 9.3%, *P*=NS 7. 16 (1.3) vs. 20.3 (1.7), *P*=.03 8a. 8.8 vs. 16.5, *P*=NS 8b. 11.8 vs. 25.9, *P*=.008 8c. 13.4 vs. 18.0, *P*=NS 8d. 17.8 vs. 18.5, *P*=NS 8e. 11.0 vs. 11.2, *P*=NS 8f. 14.8 vs. 25.6, *P*=NS 8g. 12.6 vs. 8.5, *P*=NS 8h. 12.6 vs. 9.7, *P*=NS 8i. 6.0 vs. 6.0 (LOHS available for only 1 of 2 patients in control group), *P*=NS 8j. 10.0 vs. 18.0, *P*=NS 8k. 6.5 vs. 14.0, *P*=NS 8l. 32.0 vs. (0 patients), *P*=NS 8m. (0 patients) vs. 30.0, *P*=NS 8n. (0 patients) vs. 4.0, *P*=NS 8o. 13.0 vs. (0 patients), *P*=NS 8p. 13.0 (6.9) vs. 17.6 (1.6), *P*=.01 | 0 | 0 |
| Begg, 1989[14] | **N=22 vs. 23 patients analyzed. 1. Number of patients achieving both peak (6-10 mg/L) and trough (1-2 mg/L) aminoglycoside levels at day 2 (main outcome).  2. Number of patients achieving both peak and trough aminoglycoside levels at day 5 (main outcome).  3. Number of patients achieving peak aminoglycoside levels (mg/L) in specific ranges at day 2.**  3a. > 10 (not prespecified)  **3b. 6–10 (main outcome)**  3c. 4-6 (not prespecified)  3d. < 4 (not prespecified)  **4. Number of patients achieving peak aminoglycoside levels (mg/L) in specific ranges at day 5.**  4a. > 10 (not prespecified)  **4b. 6–10 (main outcome)**  4c. 4-6 (not prespecified)  4d. < 4 (not prespecified)  **5. Number of patients achieving trough (mg/L) aminoglycoside levels in specific ranges at day 2.**  5a. 2-4 (not prespecified)  **5b. 1-2 (main outcome)**  5c. 0.5 – 1 (not prespecified)  5d. < 0.5 (not prespecified)  **6. Number of patients achieving trough aminoglycoside levels (mg/L) in specific ranges at day 5.**  6a. 2-4 (not prespecified)  **6b. 1-2 (main outcome)**  6c. 0.5 – 1 (not prespecified)  6d. < 0.5 (not prespecified)  Other prespecified outcomes 7. Mean (SEM) peak aminoglycoside level at day 2 (mg/L).  8. Mean (SEM) trough aminoglycoside level at day 2 (mg/L).  9. Mean (SEM) peak aminoglycoside level at day 5 (mg/L).  10. Mean (SEM) trough aminoglycoside level at day 5 (mg/L).  11. Mean (SEM) daily aminoglycoside dose (mg) during treatment.  12. Number of patients with dose changes (follow-up period NR). | 1. 6 vs. 0, *P*=.007  2. *P*=NS  3a. 0 vs. 0, *P*=NR 3b. 9 vs. 2, *P*=.01 3c. 7 vs. 7, *P*=NR 3d. 0 vs. 8, *P*=NR  4a. 1 vs. 0, *P*=NR 4b. 5 vs. 4, *P*=NS 4c. 4 vs. 8, *P*=NR 4d. 0 vs. 6, *P*=NR  5a. 2 vs. 3, *P*=NR 5b. 9 vs. 2, *P*=.01 5c. 5 vs. 6, *P*=NR 5d. 0 vs. 5, *P*=NR  6a. 4 vs. 2, *P*=NR 6b. 4 vs. 2, *P*=NS 6c. 2 vs. 6, *P*=NR 6d.0 vs. 2, *P*=NR  7. 6.49 (0.39) vs. 4.27 (0.52), *P*=.001 8. 1.44 (0.22) vs. 0.94 (0.21), *P*=.054 9. 7.23 (0.79) vs. 5.03 (0.46), *P*=.01 10. 1.76 (0.28) vs. 1.07 (0.15), *P*=.01 11. 312 (17) vs. 203 (13), *P*=.001  12. *P*=.15 (14 vs. 9 had no dose change; 0 vs. 4 had >3 changes). | **Prespecified**  **1. Number of deaths (follow-up period NR).  2. Change in creatinine clearance during therapy.** | 1. 1 vs. 5, *P*=.2 2. *P*=.32 (9 vs. 7 patients no change; 9 vs. 6 patients small reversible decreases; rest had small increases) | + | 0 |
| Gonzalez, 1989[22] | **Outcomes not clearly prespecified**  **1. Mean (SD) aminophylline loading dose (mg/kg) to achieve target serum theophylline level (intervention: 15mg/L, control: 10-20 mg/L).  2. Mean (SD) aminophylline maintenance dose (mg/kg/h) to achieve target serum theophylline level (intervention: 15mg/L, control: 10-20 mg/L).  3. Mean (SD) theophylline level (mg/L); baseline, 6.7 (5.2) vs. 6.8 (6.0), *P*=NS. 3a. 1 hour.  3b. 2 hours.  3c. 4 hours.** | 1. 4.2 (2.4) vs. 3.8 (2.4), *P*=NS 2. 0.6 (0.2) vs. 0.4 (0.2), *P<*.001  3a. 14.0 (2.5) vs. 12.5 (3.7), *P*=NS 3b. 14.6 (2.7) vs. 12.2 (3.8), *P*<.002 3c. 14.6 (3.1) vs. 11.4 (3.9), *P<*.001 | **Outcomes not clearly prespecified 1. Patients discharged from ED within 8 hours (i.e., not admitted to hospital).  2. Proportion of patients with adverse effects (nausea and vomiting) in ED.**  **3. Peak flow rate throughout the study** | N rand = 82; analyzed 37 vs. 30 (Number of patients NR, only %).  1. 52% vs. 47%, P<.7 2. 10% vs. 7%, P<.7 3. values not given, did not differ | + | 0 |
| Hickling, 1989[21] | **Prespecified**  **1. Number (proportion) of patients outside of therapeutic range (6-10 mg/L for peak and <2 mg/L for trough) at 48-72 hours (and who required dose change).**  **2. Mean (SEM) peak plasma aminoglycoside levels at 48-72 hours (mg/L).**  **3. Mean (SEM) trough levels at 48-72 hours (mg/L).**  **4. Number (proportion) of patients with 48-72 hours peak plasma levels:**  **4a. >5 mg/L.**  **4b. >6 mg/L.**  **4c. >7 mg/L.** | 1. 5/13 (38%) vs. 11/14 (78%), *P<*.001 2. 7.45 (0.4) vs. 5.14 (0.36), *P*<.001 3. 1.58 (0.27) vs. 0.87 (0.155), *P*=.02 4a. 13/13 (100%) vs. 8/14 (57%), *P*=.03 4b. 12/13 (92%) vs. 3/14 (21%), *P*<.001 4c. 8/13 (61%) vs. 0/14 (0%), *P*=.002 | **Prespecified**  **1. Mean increase in estimated creatinine clearance during recovery.**  Not specified  2. Number (proportion} of patients with increase in creatinine clearance at end of treatment. | 1. 17.5% vs. 20.5%, *P*=NS  2. 7/13 (54%) vs. 9/14 (64%), *P*=NS;  Of 13 in intervention group: 1 = no change, 1 = 7% decrease, 3 = 25-50% decrease, 1 unaccounted for. Of 14 in control group: 1 = no change, 4 = 0-25% decrease | + | 0 |
| Carter, 1987[17] | **1. For patients who achieved a stable PT ratio before discharge, the mean (SD) number of days from administration of the first warfarin dose to achievement of the stabilization dosage (prespecified)**  2. Number, proportion, of patients with stable PT before or at hospital discharge (not prespecified).  3. mean (SD) stabilization warfarin dosage (mg/day) (not prespecified)  4. proportion of PT ratios within each PT ratio category as measured between the time of the third warfarin dose and either achievement of a stable PT ratio or discharge (not prespecified).  4a. PT ratio ≤1.3  4b. PT ratio 1.31-2.0  4c. PT ratio 2.01-2.5  4d. PT ratio ≥2.5 | Analog vs. Linear vs. Empiric  1. 6.8 (1.26) vs. 7.33 (2.06) vs. 8.42 (3.47), *P*=NS  2. 20/31, 64.5% vs. 15/22, 68.2% vs. 19/34, 55.9%  3. 7.16 (4.41) vs. 7.44 (2.6) vs. 7.82 (3.2)  4a. 2.4% vs. 9.6% vs. 13.1% 4b. 88.3% vs. 63.8% vs. 81.7% 4c. 6.7% vs. 24.5% vs. 5.2% 4d. 0.8% vs. 2.1% vs. 0%  * No statistical analyses provided for these measures. | **1. mean (SD) time to discharge in patients without stable PT (not prespecified)** | Analog / Linear / Empiric  1. 6.3 (1.3) / 7.7 (3.5) / 6.5 (1.2)  * No statistical analyses provided for these measures. | 0 | ... |
| White, 1987[32] | **Prespecified 1. Mean (not clear if SD or SE) time to reach a stable therapeutic dose (days).  2. Mean time to reach a therapeutic PR (days).  3. n/N patients with PR above therapeutic range during hospital stay.  4. Mean predicted/observed PR.  5. Mean absolute error (absolute value of absolute PR – predicted PR).**  Not prespecified  6. % mean absolute error. Not prespecified 7. Mean days on warfarin with PR in therapeutic range during hospital stay.  8. Mean days on warfarin with PR above therapeutic range during hospital stay.  9. Mean days on warfarin with PR below therapeutic range during hospital stay  10. n/N patients reaching PR therapeutic range after 6 days [RR, 95% CI].  11. n/N patients reaching a stable therapeutic dose after 10 days.  12. Mean warfarin dose at discharge  13. n/N (%) patients with PR in therapeutic range 10-14 days after start of maintenance dose.  14. n/N patients with PR above therapeutic range 10-14 d after start of maintenance dose  15. n/N patients with PR below therapeutic range 10-14 d after start of maintenance dose  16. Mean time on warfarin (d),  17. n/N (%) patients discharged on warfarin <5.0 mg/d. | 1. 5.7 (1.7) vs. 9.4 (5.2), *P*=.002 2. 3.2 (1.6) vs. 4.5 (3.4), *P*=.05 (Note 1)  3. 2/39 vs. 6/36, *P*=.11  4. 1.75 (0.2)/ 1.76 (0.3) vs. 1.67 (0.1)/ 1.94 (0.9), *P*=NS 5. 0.20 (0.2) vs. 0.62 (0.7), *P*=.05 (Note 1)  6. 13% (14) vs. 30% (19), *P*=.05 (Note 1)  7. 58% (23) vs. 42% (27), *P*=.001  8. 3.0% (9) vs. 5.9% (14), *P*=NS 9. 39% (24) vs. 51% (31), *P*=NS 10. 1/39 vs. 6/36; 0.15 (0.02 to 0.91) 11. 0/39 vs. 11/36  12. 5.9 mg/d vs. 7.1 mg/d 13. 28/33 (85%) vs. 11/26 (42%), *P*=.002 14. 2/33 vs. 8/26 [Note: text and table data reversed for this outcome] 15. 3/33 vs. 7/26 [Note: text and table data reversed for this outcome] 16. 8.9 (6.8) vs. 11.3 (8), *P*=NS 17. 12/33 (36%) vs. 4/26 (15%)  Note 1: Author indicates *P*<.05 as significant but reports this comparison as significant. Unable to confirm with author. | **Prespecified**  **1. Mean (not clear if SD or SE) LOHS (days).**  **2. n/N patients with in-hospital bleeding complications (major/minor) during hospital stay.**  **Not prespecified.**  3. n/N deaths.  4. n/N patients with thromboembolic complications on warfarin therapy. | 1. 13 (8) vs. 20 (15), *P*=.01 2. 0/39 vs. 1(2)/36, *P*=NS 3. 0/39 vs. 0/36 4. 0/33 vs. 0/26 | + | + |
| Hurley, 1986[24] | **Prespecified 1. Patients with theophylline levels above therapeutic range (10-20 µg/mL) on days 1 and 2.  2. Patients with theophylline levels below therapeutic range (10-20 µg/mL) on days 1 and 2. 3. Patients with trough theophylline levels in therapeutic range during oral therapy.**  **4. Mean (SD) serum theophylline levels (µg/mL) Day 1**  **Day 2**  **5. Mean (SD) 1st serum level during oral therapy (µg/mL): 6. Mean (SD) trough levels during oral therapy (µg/mL).**  Not prespecified 7. Mean (SD) IV aminophylline infusion rate (mg/kg IBW/h) Day 1  Day 2 8. Mean (SD) IV aminophylline infusion duration (hours)  9. Mean (SD) hydrocortisone dose, day 1  10. Number (proportion) of patients given hydrocortisone + prednisolone during admission. | 1. day 1 *P*=NS  day 2 18.9% vs. 37.8%, *P*=.04  2. day 1 3/47 vs. 4/41,  *P=NS (*Yates-corrected Chi² = 0.035519 P = .8505*, calculated by Research Associate)*  day 2 4/37 vs. 1/37, *P=NS (*Yates-corrected Chi² = 0.857971 P = .3543, calculated by Research Associate*)*  3. 71.1% vs. 44.4%, *P*=.02  4. day 1 14.9 (3.5) vs. 15.8 (6.1), *P*=NS overall, *P*<.01 for variance  day 2 16.1 (5.2) vs. 17.9 (7.0), *P*=NS overall, *P*<.05 for variance.  5. 12.9 (4.7) vs. 10.8 (4.6), *P*=.03  6. 12.6 (3.9) vs. 9.9 (4.1), *P*=.009  Not specified  7. day 1. 0.70 (0.21) vs. 0.68 (0.15), *P*=NS overall, *P*<.05 for variance  day 2. 0.78 (0.33) vs. 0.67 (0.19), *P*=NS, *P*<.01 for variance  8. day 1. 24.0 (3.0) vs. 22.8 (4.4), *P*=NS overall, *P*<.05 for variance  day 2. 22.4 (5.4) vs. 22.1 (5.5), *P*=NS overall, *P*<.01 for variance  9. 725 (339) vs. 792 (292), *P*=NS  10. 36/48, 75% vs. 33/43, 76.7% | **Prespecified**  **N = 48 vs. 43; Other than death, # patients NR for outcomes 2 and 3, only %.**  **1. Mean peak expiratory flow rate (day 1, day 2, day 3). 2. Patients with air flow obstruction symptoms during hospitalization; data NR for all days.**  **2a. Severe breathlessness (%, day 2 and day 3).**  **2b. Wheeziness (NR by day).**  **2c. Night wheeze (NR by day).**  **2d. Cough (NR by day). 3. Patients with side effects during hospitalization; data NR for all days.**  **3a. Severe palpitations, day 2 & day 3.**  **3b. Nausea (NR by day).**  **3c. Tremulousness (NR by day).**  **3d. Agitation (NR by day).**  **3e. Blurred vision (NR by day).**  **3f. Diarrhoea (NR by day).**  **3g. Deaths (n) during 6.3-8.7 days hospitalization.**  Not prespecified  4. Mean (SD) days in hospital. | 1. Higher for intervention patients (data shown only in figure), day 1 *P*=.07; day 2 *P*=.01; day 3 *P*=.09 2a. day 2 31% vs. 48.7%, *P*=.045; day 3 16.6% vs. 50%, *P*=.01 2b. *P*=NS (no data reported).  2c. *P*=NS (no data reported).  2d. *P*=NS (no data reported). 3a. day 2 31% vs. 66.7%, *P*=.003; day 3 16.6% vs. 56.2%, *P*=.001 3b. *P*=NS (no data reported).  3c. *P*=NS (no data reported).  3d. *P*=NS (no data reported).  3e. *P*=NS (no data reported).  3f. *P*=NS (no data reported). 3g. 0 vs. 2  4. 6.3 (4.5) vs. 8.7 (6.7), *P*= 03 | 0 | 0 |
| Rodman, 1984[27] | **Main outcome: plasma llidocaine levels in middle of therapeutic range (1.5 to 5.0 μg/mL).**  **1. Mean plasma lidocaine level (μg/mL) at intervals after initiation of therapy:**  **1a. mins 0 to30**  **1ai. mins 0 to10**  **1aii mins 11-30**  **1b. mins 31 to 60**  **1c. mins 61 to 120**  **1d. hours 4 to 8**  Not prespecified  2. mean (SEM) observation time (hours)  3. mean (SEM) overall lidocaine infusion rate (μg/kg/min)  4. mean (SEM) final infusion rate (μg/kg/min)  5. mean (SEM) first-hour infusion rate (μg/kg/min)  6. number (proportion) of patients requiring upward adjustment of lidocaine to control arrhythmia in the first six hours of therapy | 1a. 2.34 vs. 1.44, *P*<.02  1ai. *P*<.3  1aii. *P*<.01  1b. 3.2 vs. 1.60, *P*<.01  1c. 3.7 vs. 2.1, *P*<.01  1d. 4.5 vs. 3.0, *P*<.01  2. 10.1 (2.0) vs. 11.3 (1.75) (NS) 3. 39.68 (7.03) vs. 35.63 (4.22) (NS) 4. 29.24 (5.31) vs. 31.24 (2.29) (NS) 5. 82.68 (6.05) vs. 42.27 (3.86) (*P*<.01) 6. 4/11 (36%) vs. 1/9 (11%) (NS) | *No outcomes were specifically prespecified.  **1. Number of patients with a toxic response requiring** lidocaine discontinuation or dosage reduction. | (N rand = 9 vs. 11) 1. 0 vs. 0 | + | 0 |

Abbreviations: AMI, acute myocardial infarction; BG, blood glucose; CCDSS, computerized clinical decision support system; CI, confidence interval; DS, decision support; ED, emergency department; FEV1, forced expiratory volume in 1 second; ICD, International Classification of Diseases; ICU, intensive care unit; INR, international normalized ratio; IQR, interquartile range; IV, intravenous; LOHS, length of hospital stay NR, not reported; NS, not significant NSAID, non-steroidal anti-inflammatory drug; OR, odds ratio; PCC, paediatric care clinic; PR, prothrombin ratio; PT, prothrombin time; PTCA, Percutaneous transluminal coronary angioplasty; RR, relative risk; SBO, small bowel obstruction; SD, standard deviation; SE, standard error; SEM, standard error of the mean; SP, Skagit paediatrics; TPI, thrombolytic predictive instrument.

^a^Outcomes are evaluated for effect as positive (+) or negative (−) for CCDSS, or no effect (0), based on the following hierarchy. Outcomes in bold font were assessed for effect. An effect is defined as ≥ 50% of relevant outcomes showing a statistically significant difference (2*P* < .05):

- If a single primary outcome is reported, *in which all components are applicable*, this is the only outcome evaluated. (see Methods section of manuscript for definition of primary outcome).
- If > 1 primary outcome is reported, the ≥ 50% rule applies and only the primary outcomes are evaluated.
- If no primary outcomes are reported (or only some of the primary outcome components are relevant) but overall analyses are provided, the overall analyses are evaluated as primary outcomes. Subgroup analyses are not considered.
- If no primary outcomes or overall analyses are reported, or only some components of the primary outcome are relevant for the application, any reported prespecified outcomes are evaluated.
- If no clearly prespecified outcomes are reported, any available outcomes are considered.
- If statistical comparisons are not reported and data are insufficient to conduct analyses, ‘effect’ is designated as not evaluated (…).

^b^Study included in 2 categories.
