# Supplementary material for: Computerized clinical decision support systems for acute care management: A decision-maker-researcher partnership systematic review of effects on process of care and patient outcomes
Source: Implement Sci. 2011 Aug 3;6:91. doi: 10.1186/1748-5908-6-91 (PMC3169487; doi:10.1186/1748-5908-6-91)
Supplement: Additional file 5 — Costs and CCDSS process-related outcomes for trials of acute care management. Cost and CCDSS process-related outcomes for the included studies. [file 1748-5908-6-91-S5.DOCX]

**Additional file 5, Table S5. Costs and CCDSS process-related outcomes for trials of acute care management ^a^**

| **Study** | **CCDSS adverse effects** | **Costs ^b^** | **Group comparison for CCDSS workflow** | **Practitioner satisfaction with CCDSS system** |
| --- | --- | --- | --- | --- |
| **Management Assistants – Alerts and Reminders** | | | | |
| Kroth, 2006[39] | … | Installation of the bedside computer workstations and vital signs monitors throughout the hospital's non-critical care area ward beds cost approximately $500,000. | … | Authors did not evaluate this directly but noted that, based on general observation, the nursing staff seemed to generally like the new system. |
| Rood, 2005[34] | ... | ... | ... | Author comment: Not described but yes [a majority of practitioners using the CCDSS were satisfied with the system]. |
| Zanetti, 2003[47] | 1-Inappropriate activation of the system, n, %. 4/449 procedures (1%).  2- Unnecessary intraoperative redosing, n=1. | ... | ... | ... |
| Overhage, 1997[26] | ... | Mean hospital charges for intervention vs. control: $8,073.52 vs. $8,589.47 (difference -$515.95, 95% CI -828.41 to 1,316.85, *P* = .68). | ... | ... |
| **Management Assistants – Guidelines and Algorithms** | | | | |
| Paul, 2006[40] | …. | 1. Cost of antibiotic treatment for all 2326 patients (intervention vs. control) in Israel; Germany; Italy; overall n (%) *P* value:  a. direct cost in Euros, mean (SD)/patient. 25.2(33.2) vs. 25.5(30.9), *P* = .079; 68.9(75.6) vs. 73.5(85.4), *P* = .674; 79.1(87.7) vs. 84.9(83.9), *P* = .302; 37.9(54.2) vs. 40.2(57.6) *P* = .473  b. observed side effect cost in Euros, mean (SD)/patient. 98.3(1048.6) vs. 88.5(1046.9), *P* = .163; 129.2 (1294.4) vs. 189.8 (1765.5), *P* = .526; 74.6 (992.2) vs. 24.4 (159.2), *P* = .819; 100.1(1085.1) vs. 99.5 (1154.0), *P* = .960  c. Ecological costs in Euros, mean (SD)/patient. 445.(404.7) vs. 511.7(439.9), *P<*.001; 517.8(374.6) vs. 503.8 (336.7), *P* = .870; 317.2 (282.2) vs. 372.2(248.3), *P* = .03; 439.5(388.4) vs. 499.3(414.1), *P* = .002  d. total antibiotic cost in Euros, mean (SD)/patient. 546.0(476.7) vs. 612.5(507.7), *P* = .001; 712.1(532.6) vs. 716.1(522.1), *P* = .960; 487.5(419.5) vs. 540.0(371.5), *P* = .135; 565.5(483.4) vs. 623.2(502.2), *P* = .01  (Note: See Appendix 2 of original paper for cost analysis calculations) | ... | ... |
| Wyatt, 1989[33] | Author comment: Yes they were - I measured false positive & false negative admissions to critical care unit + extra delays due to ACORN system use in the A&E - see my Oxford DM thesis 1992 for details. | 1- Time to collect and process data before study: Median 3 minutes for A&E nurse to complete a questionnaire; median 4 minutes for study nurse to enter data and obtain ACORN system report.  2- Time for ACORN system to produce conclusions: 5 minutes.  3- Extra delay for casualty doctors to fill out electrocardiography report: median 7 minutes | ... | ... |
| **Diagnostic Assistants** | | | | |
| Stengel, 2004[45] | ... | ... | The “workflow” measures were better with the CCDSS but the differences were not significant.  1. Integration into daily routine (4 raters for electronic documentation vs. 4 raters for conventional documentation, *P* value) - 1="very practical", 5="not practical at all"  1a. integration into daily routine.  2,2,3,2 vs. 3,3,3,2 *P* = .16  1b. time consumption. 2,3,2,2 vs. 5,2,2,2 *P* = .48  1c. handling 2,3,2,2 vs. 3,4,2,3 *P* = .15 |  |
| **Medication Dosing Assistants** | | | | |
| Cavalcanti, 2009[49] | ... | ... | 38.4% of the nurses reported that the Leuven protocol was difficult or very difficult; 13.3% reported that conventional treatment was difficult or very difficult and 11.7 % found CAIP difficult or very difficult (*P* = .78 for CAIP vs. conventional treatment; *P<*.001for CAIP vs. Leuven) |  |
| Casner, 1993[18] | …. | ... | ... |  |
| Burton, 1991[16] | ... | Costs (US $)  1. Per-patient cost avoidance (based on average bed cost and average length of stay). $1,311.45  2. Potential benefit/cost ratio (based on 6% discount & pharmacokinetic dosing service cost of $297.23/patient). 4.09:1.00 (i.e., $4.09 saved in hospitalization costs for every $1 invested in use of intervention). | ... |  |

Abbreviations: A&E, accident and emergency; ACORN, Admitted to the CCU OR Not; CAIP, computer-assisted insulin protocol; CCDSS, computerized clinical decision support system; CI, confidence interval; SD, standard deviation.

^a^ Ellipses (…) indicate outcome was not assessed.

^b^ Costs include workflow measures such as time to process alerts if these are not directly compared between groups.
